# How to design virus containment policies? A joint analysis of economic and epidemic dynamics under the COVID-19 pandemic

**DOI:** 10.1007/s11403-022-00369-2

**Published:** 2022-10-28

**Authors:** Alessandro Basurto, Herbert Dawid, Philipp Harting, Jasper Hepp, Dirk Kohlweyer

**Affiliations:** 1 Bielefeld Graduate School of Economics and Management (BiGSEM), Bielefeld University, Bielefeld, Germany; 2 ETACE and Center for Mathematical Economics, Bielefeld University, Bielefeld, Germany; 3 ETACE, Bielefeld University, Bielefeld, Germany

**Keywords:** COVID-19, Economic loss, Containment policy, Variance of policy effects, Agent-based modeling, C63, E17, H12, I18

## Abstract

We analyze the impact of different designs of COVID-19-related lockdown policies on economic loss and mortality using a micro-level simulation model, which combines a multi-sectoral closed economy with an epidemic transmission model. In particular, the model captures explicitly the (stochastic) effect of interactions between heterogeneous agents during different economic activities on virus transmissions. The empirical validity of the model is established using data on economic and pandemic dynamics in Germany in the first 6 months after the COVID-19 outbreak. We show that a policy-inducing switch between a strict lockdown and a full opening-up of economic activity based on a high incidence threshold is strictly dominated by alternative policies, which are based on a low incidence threshold combined with a light lockdown with weak restrictions of economic activity or even a continuous weak lockdown. Furthermore, also the ex ante variance of the economic loss suffered during the pandemic is substantially lower under these policies. Keeping the other policy parameters fixed, a variation of the consumption restrictions during the lockdown induces a trade-off between GDP loss and mortality. Furthermore, we study the robustness of these findings with respect to alternative pandemic scenarios and examine the optimal timing of lifting containment measures in light of a vaccination rollout in the population.

## Introduction

The ongoing COVID-19 pandemic has caused a global health crisis resulting in more than 180 million reported cases and 3.800.000 casualties, as of the end of June 2021. Policymakers in many countries have responded to the pandemic by introducing a large variety of containment measures (see Cheng et al. 2020; Haug et al. 2020). Many of these measures have substantial implications for economic activity confronting policymakers with a trade-off between a rapid containment of the pandemic and the prevention of severe economic disruptions. Finding a balanced policy mix to resolve this trade-off is a major political challenge, for which it is crucial to develop a thorough understanding of the joint epidemic (number of infected, mortality) and economic (GDP loss, sectoral unemployment) effects of different measures (Murray 2020). Whereas well-established epidemiological models can be employed to address the first of these issues (e.g., Kissler et al. 2020; Giordano et al. 2020; Ferretti et al. 2020; Britton et al. 2020), rigorous approaches for studying both dynamics simultaneously are still sparse. Considering these two aspects in an integrated framework is important not only because many containment measures have direct economic effects, but also because several main infection channels are directly related to economic activity (Chang et al. 2021).

The growing economic literature investigating the COVID-19 pandemic on a theoretical level mainly builds upon the standard equation-based SIR model to model the infectious disease ( Kermack and McKendrick 1927; Hethcote 2000) and introduces some links to economic activity. Measures taken to contain the pandemic thereby typically reduce production potential or consumption and hence induce an economic shock. The interplay between containment measures and economic costs is then studied as an optimization problem from a social planner’s point of view (e.g., Alvarez et al. 2020; Miclo et al. 2020; Acemoglu et al. 2020), or embedded in a simple macroeconomic framework, where agents individually optimize their decisions (e.g., Eichenbaum et al. 2021; Krueger et al. 2020; Jones et al. 2020). Such abstract models rely on deterministic representations of the virus dynamics and do not capture the local and complex social interactions associated with economic activities (Epstein 2009), which play an important role in the propagation of the coronavirus (see, e.g., Wu et al. 2020; Prather et al. 2020). Hence, these models neither take the interplay between economic structure (e.g., size and sectoral distribution of firms) and the transmission dynamics into account nor capture the stochastic variation of economic and epidemic dynamics. Although there exists a wide range of stochastic SIRD-type epidemic models, these approaches have not been incorporated into economic models so far.^1^

The main contribution of this paper is to examine the economic and epidemic effects of lockdown measures using a calibrated micro-founded stochastic macroeconomic model, which explicitly captures the role different economic activities play with respect to the spread of the coronavirus. In particular, our model captures virus transmissions at the workplace, transmissions caused by interactions between consumers and producers, and transmissions via private contacts. Furthermore, we consider an age-structured population, allowing us to capture age-specific differences with respect to economic activities (e.g., working vs. retired population) and the case fatality rate of COVID-19. In addition to capturing relevant transmission channels, the detailed representation of socioeconomic interaction structures allows us to implement a wide range of specific containment measures in our framework. The model is calibrated based on German micro- and macro-data and is capable of matching to empirical time series, both for economic and for epidemiological indicators, under a policy scenario resembling measures implemented in Germany. Based on this, we investigate different lockdown scenarios by systematically varying key parameters governing the intensity of measures during a lockdown, the degree of relaxation after the lockdown, and the incidence thresholds used to end/reintroduce the lockdown measures.

We show that a policy combining strict lockdown measures with a full opening-up of the economy between lockdowns and a high incidence threshold^2^ for (re)entering lockdowns is strictly dominated by alternative policies, which either combine a substantially smaller incidence threshold with weaker lockdown measures or implement a continuous light lockdown with only minor restrictions.^3^ The reason that policies alternating between strict lockdowns and full opening perform worse not only with respect to the expected number of casualties, but also with respect to economic losses, is that they induce a higher degree of volatility into the economy. In light of frictions on the labor and product market, this generates higher economic losses compared to scenarios where weaker restrictions induce only smaller, although more persistent, downturns. Similar to others (Acemoglu et al. 2020; Alvarez et al. 2020; Atkeson 2020), we find that there exists a trade-off between economic losses and infection numbers when varying lockdown intensity given a fixed incidence threshold. We also demonstrate that the policies differ substantially with respect to the ex ante uncertainty about the induced economic loss. In particular, the policies which are at the efficiency frontier also tend to give rise to substantially lower variation. Understanding the implications of different policy choices for the variance of policy results seems particularly important in an area like virus containment, where the effectiveness of chosen measures also depends on the policies’ acceptance by the general public. In such a setting, bad initial outcomes might have a detrimental effect on public acceptance of the policy, deteriorating its future effectiveness (Bargain and Aminjonov 2020; Altig et al. 2020). To our knowledge, this paper is the first economic analysis of lockdown policies, explicitly addressing the relationship between policy properties and variance of the resulting economic and epidemiological dynamics.

Since the value of the infection probability of the virus on the one hand is crucial for the epidemic dynamics and on the other hand entails a considerable uncertainty around its exact value, we conduct a robustness check of our results concerning this parameter. The main setting of our analysis is based on the assumption that at a certain point in time, the virus mutates to a more infectious variant, which then spreads in the population simultaneously with the original version. This pattern resembles the situation in many countries during the COVID-19 pandemic. We show that most of our qualitative insights, in particular the appeal of a policy combining a strict lockdown with a weak opening, still apply in scenarios without a mutation (reducing the infection probability) and with a higher infection probability. Clearly, a higher (lower) infectiousness of the virus leads to higher (lower) mortality rates and in consequence also to higher (lower) economic losses. By design, only the continuous weak lockdown is characterized by a stable loss of GDP and becomes more attractive, the higher the infection probability of the virus or its mutation. In the final part of our analysis, we assess the optimal ending point of the policies under a dynamic vaccination rollout. Depending on the lockdown policy, we find that after a vaccination rate of $25-40\%$ no significant gains in terms of mortality are achieved when prolonging the containment measures. We conclude that in our setting, lockdown policies can be lifted relatively early.

In light of the mechanisms underlying our insights, our qualitative results can be transferred to countries with a health system and economic structure comparable to Germany. In addition, the flexibility of the framework allows the modeler to adjust the parameters related to COVID-19 to analyze potential future pandemics. In fact, the model can easily be re-calibrated to data from other countries or from different pandemics in order to analyze appropriate policies under alternative structural conditions.

Methodologically, our approach combines a SIR-type simulation model with an agent-based macroeconomic model. The design of the economic part of the model, in particular with respect to the structure of the individual decision rules as well as the market interaction protocols, builds strongly on a well-established agent-based macroeconomic framework, namely the Eurace@Unibi model, which has been already used for the analysis of a wide range of economic policy issues (e.g., Dawid et al. (2014, 2018, 2019)). Nevertheless, the model employed here is not an extension of the Eurace@Unibi model, but a separate agent-based model designed for the analysis of the interplay of economic activities and virus transmission, which has also been implemented independently from the Eurace@Unibi model.^4^

Agent-based models have been used to assess the effectiveness of containment policies in purely epidemiological studies (e.g., Adam 2020; Ferguson et al. 2020; LeBaron 2021) and the approach has been applied to address a large variety of macroeconomic research questions and policy analyses in recent years (see, e.g., Foley and Farmer 2009; Dawid and Delli Gatti 2018; Dosi and Roventini 2019, for discussions). By explicitly linking economic activities and transactions to contacts between agents, agent-based economic models are particularly suited for studying the dynamics of virus transmissions in an economy. Only a few other studies have used a unified agent-based model, combining an economic framework with an epidemiological structure in the context of COVID-19. Delli Gatti and Reissl (2020) opt for a relatively smaller model in terms of the number of agents with 2800 households and 300 firms calibrated on an Italian region, Lombardy. Our paper differs from theirs in terms of the main research question. While their focus is on the consequences of different fiscal measures, our main goal is to understand the impacts of several lockdown policies on economic loss and mortality while keeping in place the fiscal measures introduced by the German government. Moreover, we also introduce the occurrence of a virus mutation in a macro-epidemic agent-based model. Sharma et al. (2021) introduce the COVID-19 crisis as exogenous shocks of demand and supply, dropping firm productivity and household consumption propensity, into the Mark-0 agent-based model. Shocks amplitude and duration are the main factors describing the crisis but epidemiological dynamics is not considered. Mandel and Veetil (2020) use a multi-sector disequilibrium model with an agent-based flavor to study the impact of COVID-19 related lockdowns, taking into account input–output data and supply chain effects. However, this paper also does not consider epidemiological dynamics. Mellacher (2020) has a very rich network-based model that enables a detailed treatment of contact spaces such as households, hospitals, or retirement homes. On the other hand, it does not feature shopping contacts which we consider crucial to assess the interconnection of economic and pandemic effects of closures. Silva et al. (2020) present a model to study the effects of various counterfactual containment scenarios on a virtual economy representing Brazil. However, our paper is the first to use such a unified framework for the evaluation of average economic and pandemic effects as well as the associated uncertainty about outcomes under different policy responses to the outbreak of the COVID-19 pandemic.

The paper is organized as follows. In Sect. 2, we provide a short description of the model (a detailed description is given in Appendix A), and in Sect. 3 we describe the set of containment policies considered in our analysis. In Sect. 4, we discuss the calibration of the model and demonstrate the good fit of the model output with time series data from Germany. The main insights from our policy analysis are discussed in Sect. 5, and we end with conclusions and an outlook on potential extensions of the analysis in Sect. 6. In addition to the detailed description of the model, the Appendix contains some results with respect to additional policy variations, statistical test results underlying our findings, and lists of model variables and of parameter values.

## The model

In this section, we provide a short description of our model, which highlights the overall structure of the economy as well as the crucial assumptions and mechanisms driving the economic and pandemic dynamics. A more detailed and technical presentation of the model is given in Appendix A.

### Economy

**Fig. 1:**
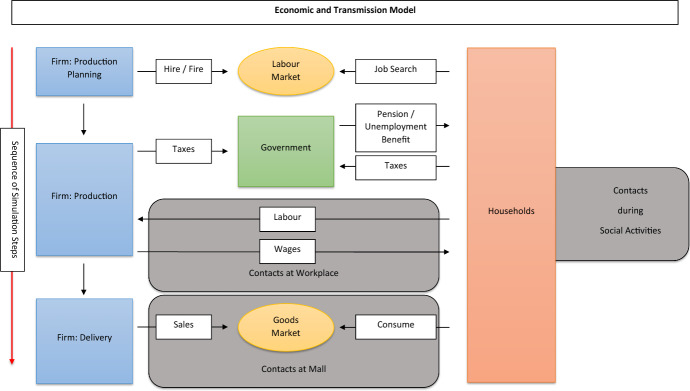
Summary of model structure

The economy consists of firms as well as young and old households. Young households constitute the labor supply of the economy, whereas old households live on a pension that is paid through a pay-as-you-go system. There are three private and one public sector in the economy. We explicitly represent these different sectors in the model in order to be able to capture sectoral differences with respect to firm size and the number of contacts a household has by consuming a product of a particular sector, as well as to analyze the effects of sector-specific reductions in consumption and economic activity due to lockdown measures.^5^ The basic time unit in our model is one day and activities of agents take place daily or periodically, e.g., once a week (household consumption, firm production planning, etc.). The behavioral rules determining the actions of firms and households are based on the literature on macroeconomic agent-based modeling and, in particular to a large extent, resemble the corresponding rules developed for the Eurace@Unibi model (see Dawid et al. (2019)). Figure 1 illustrates the overall model structure.

#### Firms

Firms are distributed across three private sectors: manufacturing (M), service (S), and food (F), where the latter represents all essential products for daily life. A firm *i* is characterized by a firm-specific productivity level $A_i$ and employs $L_{i,t}$ workers in period *t* to produce a weekly output $Q_{i,t}$ according to the linear production function $Q_{i,t} = A_i \; L_{i,t}$.^6^ The firm’s activity and planning cycle is one week and each firm plans and carries out its production at the beginning of each week. The produced quantity replenishes the firm’s inventory stock at the sector-specific mall. At the mall, all producers in that sector offer their products at posted prices. In particular, the firm carries out the following steps:

1. The firm determines the target level of inventory at the beginning of the week based on its adaptive demand expectation and the size of a sector-specific safety buffer, which is determined based on estimated demand volatility.
  The resulting planned production quantity determines the desired size of the firm’s workforce. If the size of the firm’s current workforce is larger than the desired number of employees, the firm dismisses the appropriate number of randomly picked workers.
2. If the firm needs to increase its workforce it opens vacancies and unemployed job seekers skilled to work in the firm’s sector apply. Firms announce their openings in random order and hire on a first-come-first-serve basis. If there are no job seekers at the time of the announcement, the firm is rationed and can only hire again in the following week.
3. Production of output $Q_{i,t}$ takes place. Products are offered in the sector-specific mall at posted prices. Firms set prices $p_{i,t}$ by applying mark-up pricing with an endogenous mark-up on unit costs. The mark-up adaptively evolves over time within a fixed interval and depends positively on the firm’s market share.
4. Firms pay wages $w_i$, which are sector-specific and proportional to the average productivity in the sector, as well as taxes and dividends. Dividends are determined as a fraction $\zeta$ of net profits, where $\zeta =1$ if the firm’s liquidity exceeds a threshold and $\zeta < 1$ otherwise. Dividends and the fixed costs paid by the firms are equally distributed among households to ensure the stock-flow consistency of the model. A firm with negative liquidity declares bankruptcy and exits the market. It dismisses all workers and the firms’ inventory stock is fully written off.^7^

#### Households

While old households are retired, young households are active on the labor market. Each household has appropriate skills to work in one sector of the economy. The households’ weekly activity sequence is as follows:

1. Unemployed households apply for open positions.
2. Households receive wages, unemployment benefits, pensions as well as dividends and pay taxes.
3. Households determine their consumption budget for the upcoming week according to a buffer-stock saving heuristic, see Deaton (1991). In particular, households spend exactly their weekly net income as long as their current wealth corresponds to a desired wealth-to-income ratio. Otherwise, consumption spending is adjusted such that the wealth-to-income level moves toward its target value. The consumption budget is allocated across the three sectors according to fixed (empirically determined) consumption shares. However, there is a lower bound on the factor by which the consumption budget for food/essential products is allowed to change between consecutive weeks.
4. Each household has a day of the week for each sector $k \in \{M,S,F\}$ at which she considers to visit the sector-specific mall. The household visits the mall on that day with probability $p^s_k$, where in the absence of lockdown measures $p_k^s = 1$ for all sectors *k*. Upon visiting the mall, the household scans the posted prices in a randomly chosen subset of firms in the sector and chooses the firm to buy from according to a logit choice function based on these prices. The purchased quantity is determined by the household’s consumption budget for that sector.
  If the inventory of a firm at the mall becomes zero during a week, the firm is no longer considered by households in their consumption choice until the inventory is filled up again. If there are no active firms in the mall when a household *h* visits the mall or if the chosen firm is not able to supply to the total amount demanded, then the consumer is rationed and returns to the mall again the following day. All parts of the foreseen weekly consumption budget for sector *k* which have not been spent after that second shopping day, are added to the household’s savings.

#### Public sector

Besides the three private sectors, there is also a public sector operated by the government with a constant number of government offices. The public sector provides administrative services that are not sold on the goods market.^8^ Each public sector office employs a set of civil servants that only changes over time if an employee dies. The government collects income and profit taxes to finance the wage bill of the public employees and to pay unemployment benefits, pensions, and potentially lockdown-related transfers to individuals and firms. Unemployment benefits are based on the last wage of an unemployed worker with replacement rate $\nu$. Pensions are uniform for all old households and are determined as a constant percentage of the average wage in the economy. Households employed in the public sector earn a fixed wage $w^P$. The government adjusts the tax rate over time in order to keep the public account at a given target level.

### Virus transmission

Virus transmission is modeled by explicitly tracking contacts between agents and potential infection chains. Following a standard SIRD approach households can be in one of the four states: susceptible, infected, recovered, or deceased (see, e.g., Hethcote 2000). In the absence of any policy measures, a susceptible household is infected with the homogeneous infection probability $p_{inf}$ at each contact with an infectious agent. Due to policy measures, this probability is reduced to $(1 - \xi ) p_{inf}$, where the value of $\xi$ depends on the type of measures implemented (see below). After infection, an agent first enters a homogeneous latency period of length $t_{ltn}$, followed by a period where the agent is infectious (length $t_{inf}$). Following this, agents enter the post-infectious phase, and recover after $\bar{t}_{rec}$ periods, unless they pass away before that. While being infected, a household of age *a* dies with a case fatality rate $q_t^{a}$ with $a \in \{y,o\}$. The rate does not only depend on the age of the household, but also on the degree of utilization of intensive care units in the economy at time *t*. In particular, depending on the degree of over-utilization, the age-specific fatality rate is a weighted average between a regular fatality rate $\bar{q}_l^{a}$ achieved with under-utilized intensive care units and a fatality rate $\bar{q}_h^{a}$ that would be achieved if no intensive care capacities would be available. Formally

1 $$\begin{aligned} q_t^{a} = \left[ \frac{\min (n^{icu},u^{icu} \cdot \vert \textbf{I}_t\vert )}{u^{icu} \cdot \vert \textbf{I}_t\vert }\right] \bar{q}_l^{a} + \left[ 1-\frac{\min (n^{icu},u^{icu} \cdot \vert \textbf{I}_t\vert )}{u^{icu} \cdot \vert \textbf{I}_t\vert }\right] \bar{q}_h^{a} \end{aligned}$$

where $n^{icu}$, $u^{icu}$, $\textbf{I}_t$ are, respectively, the number of intensive care beds available, the fraction of infected individuals in need of intensive care and the set of infected agents at *t*.^9^ If a household passes away, the current savings of this household are inherited by a randomly selected living household to ensure stock-flow consistency of the model. In case the intensive care units are under-utilized, the fatality rate only depends on the agents’ age-group. If, however, the demand for intensive care units exceeds the availability, the fatality rate increases proportionally to the size of the shortfall.

#### Virus mutation

Based on empirical observations during the COVID-19 pandemic, we assume that at day $t^{mut}$ a new and more contagious virus mutation emerges. For a household infected with the new variant, the individual infection probability $p_{inf}^{mut}$ of the mutant is higher compared to the original virus, while the remaining epidemiological parameters are the same as for the original virus. At $t^{mut}$ a small number of infected households switches to the virus mutation and afterward there are two coexisting virus strains spreading across the population, where upon being infected a household inherits the type from the infecting agent.^10^

**Fig. 2:**
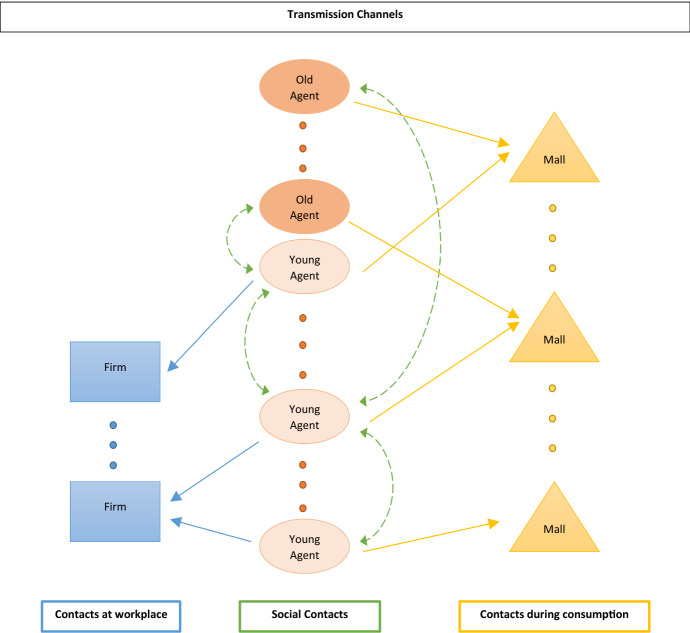
Summary of transmission channels

#### Social interactions

Contacts between households may take place on three different occasions, each potentially contributing to the propagation of the virus (see Fig. 2):

(i) Employed households have contact to a number of co-workers at their employer every day. More precisely, every worker *h* employed in sector $k \in \{M,S,F,P\}$, apart from those working from home or on short time work^11^, meets every day *t* a set of $N_{h,t}^{w}$ randomly selected colleagues employed at the same firm, respectively, public office. The number of meetings on a given day, $N_{h,t}^{w}$, is determined by independent random draws from a uniform distribution between 0 and $n_k^w$. We allow the upper bound $n_k^w, \ k \in \{M,S,F,P\}$ to vary between sectors.
(ii) During their consumption activities, households have contacts with other agents visiting the same mall on the same day. For the service sector, this also includes contacts during the consumption of a service (e.g., at a restaurant or a fitness studio). More precisely, for each sector *k* the parameter $n_{k}^c$ determines the maximum number of possible meetings on one shopping day and, similar to above, for each household visiting a mall in sector *k* at *t* a specific value ${\bar{N}}_{h,k,t}^{c}$, is drawn randomly between 0 and $n_k^c$. The actual number of people met by a household *h* visiting a mall of sector *k* is then given ${\bar{N}}_{h,k,t}^{c}$ multiplied by the ratio between the actual number of visitors to that mall and the number to be expected if an equal fraction of all households visit the mall every day of the week. This formulation captures that fewer interactions, and therefore also fewer infections occur during shopping or consumption of services if households reduce their consumption activities. Also, it allows to capture that the average number of interactions during consumption might vary substantially between sectors.
(iii) Agents also have social contacts not directly related to economic activities. We distinguish between the frequencies of intra- and inter-generational contacts for the different age-groups. In particular, the number of contacts for each type of meeting of agents across age-groups is drawn from a uniform distribution whose upper bound $n^p_{a,a'}, \ a,a' \in \{y, o\}$ reflects the cross age interaction patterns, based on empirical data.

This approach to modeling social interaction implies that the actual number of contacts for a household is stochastic with sector- and age-specific expected values that have been informed by empirical data. It should also be noted that we assume that agents do not change their behavior and their contact patterns after infection. This simplifying assumption is based on the observations that a large fraction of infected individuals do not show symptoms^12^ and that at least in the initial months of the pandemic no large-scale testing facilities were available.

## Policy measures

### Containment measures

The containment measures addressed in our policy analysis are inspired by a set of measures implemented in different countries after the outbreak of COVID-19 (Cheng et al. 2020) and can be grouped into four categories:

(i) Individual prevention measures reducing the infection probability at face-to-face contacts between an infected and a susceptible agent from $p_{inf}$ to $(1-\xi ) \ p_{inf}$, respectively, $p_{inf}^{mut}$ to $(1-\xi ) \ p_{inf}^{mut}$, with $\xi \in (0,1)$. These measures include keeping a minimum physical distance, improved measures of sanitation, and wearing face coverings.
(ii) Social distancing measures reducing social interactions in the private context either through contact restrictions imposed by the government or through a consensual change in the behavior of individuals. Studies show that there has been a substantial reduction in the number of social contacts in Germany after the outbreak of COVID-19 (e.g., Lehrer et al. 2020). In our model, social distancing is captured by a reduction in the average number of daily intra- and inter-generational social contacts.
(iii) Reduction in contacts at the workplace, by allowing a sector-specific fraction $h_k^{ho}$ of employees to work from home (see Fadinger and Schymik 2020; Möhring et al. 2020).
(iv) Reduction in consumption activities, by reducing the sector-specific weekly shopping probabilities $p^{s}_k$. Such a reduction might be induced by restrictions from the government (lockdown), or by voluntary changes in individual consumption behavior due to public information about potential infection risks. More precisely, we assume that the weekly shopping probability during lockdown is reduced to
  2 $$\begin{aligned} p^{s,l} = (1,1,1) - \alpha ^l (\Delta p^{s,l}_M, \Delta p^{s,l}_S, 0). \end{aligned}$$
  The parameter $\alpha ^l$ governs the intensity of the lockdown and $\Delta p^{s,l}_M, \Delta p^{s,l}_S$ are calibrated such that the intensity of the lockdown measures taken in Germany in March 2020 corresponds to $\alpha ^l = 1$. Shopping probabilities in the food and essential goods sector are not reduced during lockdowns. In contrast to measures (i)–(iii), the reduction in consumption activities has a direct negative impact on economic activity. In order to capture policies that include partial reduction in consumption also in periods without an actual lockdown in place, we introduce a second parameter $\alpha ^o$, governing the degree of opening. The sector-specific weekly shopping probability in periods without lockdown (as long as the containment policy is active) is given by
  3 $$\begin{aligned} p^{s,o} = (1,1,1) - \alpha ^o (\Delta p^{s,l}_M, \Delta p^{s,l}_S, 0). \end{aligned}$$

### Economic support programs

We assume that economic support measures accompany the virus containment policies in order to counteract the economic disruptions and to keep the number of insolvencies low:

(i) Under the *short-time work scheme* firms put a fraction $q^{st}$ of employees on short-time work. Employees on short time receive a fraction $\varphi < 1$ of their regular wage paid by a transfer from the public account.
(ii) Under the *bailout policy*, the government bails out any firm with negative liquidity in a given period balancing the firm’s account with a transfer from the public account, thereby avoiding bankruptcy.

In our policy analysis below, we assume that both measures are activated at the time of the first lockdown, and then maintained till the containment policy is lifted. As shown in Basurto et al. (2020), the qualitative findings discussed in Sect. 5.2 carry over also to scenarios without economic support programs in place.

## Model calibration

Our basic calibration approach is to determine the values of the majority of the parameters based on direct empirical evidence from different data sources and calibrate the remaining parameters, for which no such direct evidence is available, in a way that the model output well matches empirical time series of key variables related to the pandemic and economic indicators in Germany between the outbreak of the pandemic and the end of the third quarter of 2020. Here, we provide a summary of the key aspects of this approach, and details of the calibration of the model are provided in Appendix A.4. It is based on demographic and statistical data from Germany, as well as empirical studies on age-structured social interaction patterns. We target key aggregate economic indicators, e.g., per capita GDP, unemployment rate, and the value of the $R_t$ coefficient for the coronavirus in the absence of any countermeasures.

**Fig. 3:**
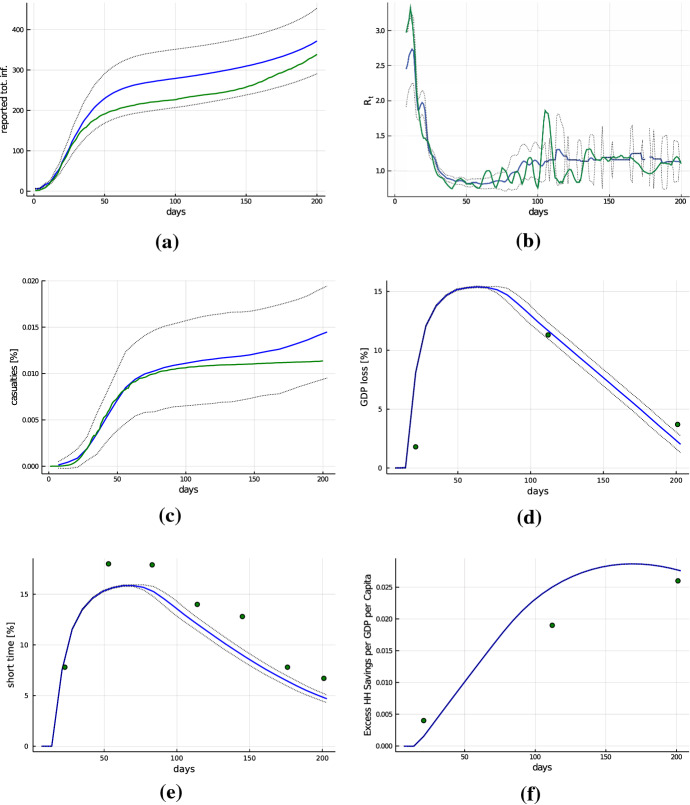
Comparison of simulation output with empirical data for Germany. Blue solid lines show the average over 50 Monte Carlo runs, black dotted lines plus–minus one standard deviation bands across Monte Carlo runs. Green solid lines show empirical counterparts based on epidemiological data from Johns Hopkins University (Johns Hopkins University 2020) for Germany from March 9, 2020 (day 0) to the end of the third quarter (day 205), scaled to a population of 100.000 inhabitants and adjusted by a detection rate. Containment and lockdown measures are introduced after 14 days into our simulation (corresponding to March 23, 2020) and are lifted on day 63 (May 11, 2020) (Cheng et al. 2020). **(a)**, Accumulated number of infected. **(b)**, weekly smoothed $R_t$ value **(c)**, casualties as a percentage of the population. **(d)**, monthly GDP loss as a percentage of the baseline GDP with green dots showing quarterly GDP loss (Eurostat 2020a) **(e)**, percentage of workers in short-time program with green dots showing estimated number of workers in short-time program relative to the size of the active labor force for April to August in Germany (Bundesagentur für Arbeit 2020). **(f)**, excess savings per household relative to baseline GDP per capita with green dots showing empirical excess savings relative to baseline GDP (ECB 2021) (color figure online)

We initialize the model with $m_0 = 100.000$ households and $n_0 = 3780$ firms (private and public). The number of households is chosen to balance the trade-off between having a sufficiently large population size and technical limitations. The total number of firms, instead, has been chosen to match the relation between the size of the working population and the number of private firms in the German economy. The initial population state we use for all our simulations has been generated by running our model for a burn-in phase of 2300 periods. Without the appearance of the virus and any change in the policy parameters, the model exhibits stationary dynamics of the economic key variables like GDP and unemployment starting from this initial state.

We use data from the German statistical office on demographic structure, sectoral distributions of productivity and consumption spending, average firm size, and the average unemployment rate. The sector-specific fractions of workers eligible for working from home are based on empirical studies by Fadinger and Schymik (2020) and Möhring et al. (2020). Parameterization of behavioral rules are taken from the well-established models in the agent-based macroeconomic literature (see, e.g., Dawid et al. 2019). Additional economic parameters, like the firms’ sector-specific inventory buffers or mark-up ranges are calibrated to generate a stationary GDP per capita and unemployment rate that reasonably match the German economy before the pandemic. In particular, for the pre-pandemic period, the model generates an annual GDP per capita of 43.013€ and an unemployment rate of $3.98\%$ (average over 50 runs) compared to an annual GDP per capita (Eurostat 2020a) of 41.350€ and an average unemployment rate (Eurostat 2020b) of $3.2\%$ in 2019.

Epidemiological parameters, like fatality rates, intensive care utilization^13^, and the detection rate, are taken from German data (mainly from the Robert Koch Institut), whereas the parameters determining the duration of latency and infectiousness after being infected are based on World Health Organization data. Our representation of a virus mutation arising after about 6 months is based on data about the ’$\alpha$-mutation’ of SARS-CoV-2 detected in Great Britain in September 2020. The (age-structured) number of social contacts associated with different activities is taken from survey studies on this topic (Mossong et al. 2008). The calibration of the parameters describing the containment measures, the sector-specific effects on consumption and on the reduction in contacts is based on German data on policy interventions, societal activities, and economic losses during and after the first lockdown in March 2020 (see Appendix A.4 for all parameter choices and sources). The individual contact infection probability $p_{inf}$ is calibrated to match an $R_0$ value of 2.5 (without any containment policy), in accordance with empirical evidence (Read et al. 2020). The effectiveness of individual prevention measures $\xi$, for which no direct empirical observations are available, is calibrated by targeting key properties of infection dynamics in Germany over a time span of 6 months. In particular, we use two separate values for this parameter: For the first lockdown phase starting on March 9, 2020, we use $\xi ^l=0.625$, and for the opening-up phase after May 11, 2020, we use $\xi ^o=0.55$. In Fig. 3, we compare the simulation output of the model under policies resembling German measures (blue) with actual German data (green) from March 9, 2020 to the end of the third quarter of 2020.^14^ Although only three free parameters ($\xi ^l, \xi ^o, q^{st}$) were calibrated to target these empirical time series, the generated data is consistent with its empirical counterpart, both with respect to levels and dynamic patterns. This applies to infections and mortality (Fig. 3a–c) as well as to the time series of economic indicators, like the GDP loss, the number of workers in short-time program, or dynamics of households’ excess savings relative to the pre-crisis level (Fig. 3d–f).

## Policy analysis

Having established the ability of our model to qualitatively and also quantitatively reproduce German epidemiological and economic time series under a policy scenario mirroring actual measures taken in Germany, we will now explore the epidemiological and economic implications of alternative policies. Our analysis begins in Sect. 5.1 with a policy scenario in which we only consider measures without direct economic effects. This part demonstrates that restricting attention to such policies is not sufficient to keep the number of infected at a level to avoid over-utilization of intensive care unit capacities. Based on this, we focus on our main analysis in Sect. 5.2 on lockdown policies that are associated with direct economic restrictions and compare the effects of different designs of such policies. In Sect. 5.3, we run the same policy analysis but first, excluding the emergence of a more contagious virus mutation and second, increasing the infection probability by $25\%$ to assess the robustness of the derived policy results. In Sect. 5.4, we investigate the optimal phase-out of our main policies under a dynamic vaccination rollout.

### Policies without direct economic impact

An important question is whether the spreading of the virus can be reduced with containment measures not directly interfering with economic activities in the sense of closing stores or reducing the possibility to consume services. In order to address this question, we consider three policy scenarios: first, a scenario where no containment measures are taken at all; second, the introduction of only individual prevention measures; and third the combination of these individual prevention measures with working from home.

**Fig. 4:**
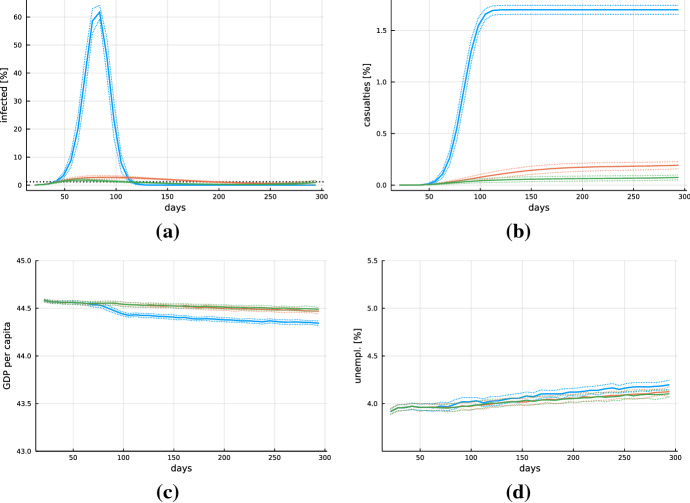
Dynamics. Solid lines show averages over 50 Monte Carlo runs, dotted lines plus-minus one standard deviation bands across Monte Carlo runs. **a** Dynamics of currently infected individuals and **b** total casualties as well as **c** GDP per capita and **d** unemployment rate for the scenarios with no policy measures (blue), only individual prevention measures (orange) and individual prevention measures in combination with working from home (green). The black dotted line in panel **a** indicates the upper bound on the number of infected under which the intensive care capacities are still not fully used (color figure online)

Figure 4 shows the dynamics of the percentages of the population currently infected and deceased. The curve of infected individuals in the absence of any measures (blue) follows a steep hump-shaped pattern well known from standard SIRD models (Hethcote 2000). Due to herd immunity, the virus is eliminated after approximately 120 days but the associated mortality is about 1.6%. This illustrates that our transmission model is producing reasonable, characteristic epidemiological dynamics.

To see the effect of individual prevention measures in our model, we analyze a setup in which only $\xi$ increases to the calibrated benchmark value of $\xi ^l = 0.625$ 2 weeks after the appearance of the virus. The introduction of the individual prevention measures (orange) strongly reduces the speed of the diffusion of the virus and the maximum number of infected. Complementing individual prevention measures with working from home (green) reinforces these effects and average mortality can be reduced by a factor of approximately 10 compared to the scenario without any containment. Nevertheless, we observe in Fig. 4a that the curve indicating the average number of infected crosses the black dotted line, which represents the upper bound of infected compatible with intensive care unit capacity, both in the scenario with only individual prevention measures as well as in the scenario where working from home is introduced in addition. Hence, these measures are not sufficient to ensure that the number of infected stays below the intensive care capacity.

Considering the GDP and unemployment dynamics shown in Fig. 4c and d, it is confirmed that these measures are not associated with any direct economic costs.^15^ A crucial assumption in this respect is that in our setting productivity of workers is not reduced when they work from home. The slight decrease in GDP and increase in unemployment around period 100 in the scenario without containment measures is due to the reduction in demand induced by the large mortality. Since the majority of deceased are old agents, who are not part of the workforce and receive their income entirely through transfers, the reduction in consumption spending induced by the death of these agents is partly offset by a reduction in income tax and the resulting increase in the consumption budget of the remaining population. The adaptation of the tax rate is, however, sluggish such that the pandemic wave nevertheless induces a contraction with some increase in unemployment, which then triggers negative follow-up effects on employment and slows further downward adjustment of GDP even after the pandemic wave is over.

**Fig. 5:**
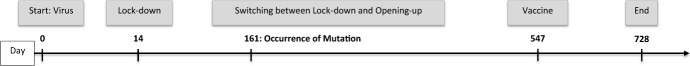
Timeline underlying the policy analysis

### Policies with direct economic impact

In the following analysis, we focus on the design of containment measures with direct economic impact. In order to compare different policies we use two main indicators: (i) virus mortality, measured as the percentage of the population deceased due to the virus 24 months after the virus outbreak and (ii) the average percentage loss in GDP (relative to the pre-virus level) during this time interval. Similar to the default policy scenario (Fig. 3), we assume that 2 weeks after the initial occurrence of the virus at $t=0$, individual preventive measures, social distancing, working from home, and lockdown measures are activated.^16^ The design of the lockdown policy is then characterized by the following three key parameters, which are systematically varied in our analysis:

(i) Intensity of the lockdown reducing the shopping probability: $\alpha ^l$ [see eq. (2)].
(ii) Reduction in weekly shopping probability in periods without lockdown if the virus is still active: $\alpha ^o$ (see eq. (3)).
(iii) Incidence threshold $\beta ^l$: the lockdown stage is re-activated if the reported number of weekly newly infected per 100.000 households rises above $\beta ^l$.

As mentioned above, the benchmark policy resembling the German scenario in Sect. 4 corresponds to $(\alpha ^l, \alpha ^o, \beta ^l) = (1,0,50)$.^17^ During the opening-up phase, we assume that the working from home measure remains active. After approximately 5 months, we introduce a virus mutation with an infection probability increased by $50\%$. Moreover, all runs are based on the assumption that 18 months after the first occurrence of the virus, a vaccine has been developed and a sufficient percentage of the population has been vaccinated to prevent any further transmissions of the virus. Hence, at that point $p_{\inf }$ and $p_{inf}^{mut}$ are set to zero and all containment measures are lifted. Since a full economic recovery might still need some time even after all restrictions have been removed, our analysis covers a time window of 24 months after the first introduction of lockdown measures. Figure 5 summarizes the timeline underlying our policy analysis. For each considered policy scenario, we carry out 50 simulation runs of the model in order to capture the variance of the emerging dynamics.

**Fig. 6:**
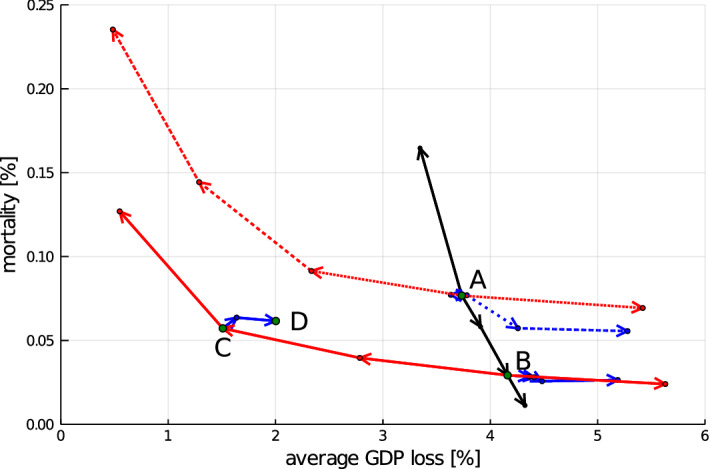
Effects of variations of key policy parameters. All points correspond to averages across the 50 runs. GDP loss [%] on the x-axis measured as loss averaged over simulation time span of 728 periods (24 months) as a percentage of baseline and mortality [%] on the y-axis expressed as a percentage of population

**Fig. 7:**
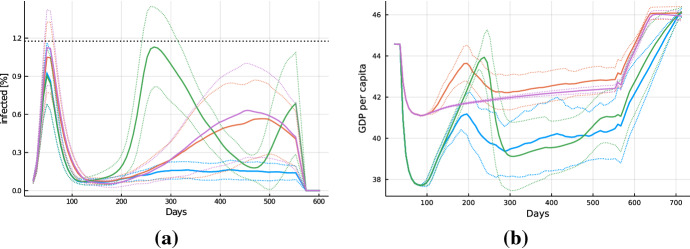
Dynamics. Evolution of the **a** current number of infected and **b** the GDP per capita for the five policy scenarios (A: green; B: blue; C: red; D: purple). Solid lines indicate batch run means and dotted lines indicate plus/minus one standard deviation (color figure online)

The main results from our analysis are summarized in Fig. 6, which shows the average GDP loss and total mortality after 24 months (mean over the 50 runs) under a systematic variation of the policy parameters. Starting from point A, corresponding to the calibrated German policy scenario (1, 0, 50), we systematically vary the key parameters $(\alpha ^l, \alpha ^o, \beta ^l)$. First, along the black line we decrease the incidence threshold $\beta ^l$ for entering a lockdown, reaching a policy (1, 0, 5) in point B, whereas for the highest point we increase $\beta ^l$ to 100. Second, along red lines (solid and dashed) we decrease (left arrow) or increase (right arrow) the intensity of the lockdown, $\alpha ^l$, with a step size of 0.25. In the following analysis we will in particular consider the policy (0.5, 0, 5), labeled as C, which combines a low lockdown threshold with weak restrictions during the lockdown. Finally, along blue lines (solid and dashed) we increase the restrictions in the opening-up phase, $\alpha ^o$, in steps of size 0.25. Hence, point D (0.5, 0.5, 5) represents a policy of continuous weak restrictions of economic activity. Figure 7 shows the dynamics of newly infected (panel a) and per capita GDP (panel b) for the four key policy scenarios corresponding to points A–D. Table 1 contains mean values and standard deviations for key indicators, such as the duration of the lockdown or the public deficit, across the batch runs for each of the four key policies. In Tables 10 and 11 in Appendix D we provide information on the statistical significance of the differences in induced GDP loss and mortality between the key policies based on Mann–Whitney U tests.

**Table 1:** Comparison of policy results

	A	B	C	D
$(\alpha ^l, \alpha ^o,\beta ^l)$	(1, 0, 50)	$(1,0,{\textbf {5}})$	$({\textbf {0.5}},0,{\textbf {5}})$	$({\textbf {0.5}},{\textbf {0.5}},{\textbf {5}})$
	*Benchmark policy*	*Low threshold*	*Weak lockdown*	*Cont. weak lockdown*
GDP loss [%]	3.73 (0.76)	4.16 (0.91)	1.51 (0.33)	2.00 (0.12)
Mortality [%]	0.077 (0.016)	0.029 (0.008)	0.057 (0.025)	0.062 (0.025)
Duration in lockdown	421.4 (135,2)	495.2 (169.4)	572.74 (151.0)	529.9 (194.9)
Number of lockdowns	2.48 (0.61)	6.40 (2.11)	4.04 (1.35)	2.76 (1.17)
Pub. Acc. Deficit [% of GDP]	5.50 (1.28)	5.18 (1.56)	2.94 (0.75)	2.31 (0.46)

Cells show means over 50 batch runs with standard deviation in brackets

Based on these Figures and Tables we derive four qualitative insights about the implication of different types of lockdown policies.

#### Result 1

Policies with a continuous ‘weak lockdown’ dominate policies with switches between strong lockdown and full opening (C, D vs A).

Figure 6 shows that policy scenario A is clearly dominated by policies C and D, which both result in lower expected values of mortality and lower GDP loss. Considering the very high average lockdown duration under policy C, it is hardly surprising that the effect of this policy is close to that of policy D, which essentially implements a weak lockdown throughout the entire 18 months in which the virus is active. As can be seen in Fig. 7 the main economic advantage of these policies is that they induce a much weaker initial downturn, compared to a policy with a strong initial lockdown (such as A). The reduction in economic activity caused by strong lockdown measures has an immediate negative impact on a firm’s production planning and household’s wage income. Hence, even after lockdown measures have been lifted, the adjustment of consumption spending and production plans needs time and in combination with (labor market) frictions this implies a relatively slow economic recovery.^18^ Therefore, under policies characterized by strong lockdown-induced downturns, accumulated GDP loss grows in a convex way with the size of these downturns. Hence, avoiding the large costs associated with a strong initial reduction in economic activity induces smaller economic losses, even if the constraints have to be preserved for an extended period of time, as in policy D. Figure 7a shows that implementing only weak lockdown measures leads to a larger initial peak and a delayed decrease in the infection numbers, compared to a strong lockdown (A). However, the continuous application of lockdown measures prevents a second wave and hence overall mortality in policies C and D is below that of A. Finally, it should be noted that, due to the induced strong and repeated economic downturns, policy A also results in a larger increase in the public deficit compared to policies C and D (Table 1). These differences in public deficit are mainly due to more extensive use of the short-time work scheme in policy scenarios A and B, whereas there is only a minor difference in bailout expenditures between the scenarios.^19^

#### Result 2

Two trade-offs emerge. For a given lockdown intensity $\alpha ^l$, decreasing the lockdown threshold $\beta ^l$ induces lower mortality but increases economic losses (A vs. B). For a given lockdown threshold $\beta ^l$, a variation of the intensity $\alpha ^l$ results in a trade-off between mortality and GDP loss (B vs. C).

In terms of infection dynamics (Fig. 7a), a higher threshold causes a visible second wave which is absent for a lower threshold. A threshold of $\beta ^l=50$ results in two lockdowns for most runs (Table 1), which are necessary in response to resurgence of the virus. In contrast, policy B with threshold of $\beta ^l= 5$ causes numerous lockdowns and accumulates a longer total duration in lockdowns over the whole time span. These repeated lockdowns keep the number of infected low, which explains the substantial reduction in mortality relative to policy A. In terms of GDP, both policies are characterized by a strong initial downturn and the trajectories only begin to deviate from each other in the recovery phase. Under a low threshold (policy *B*) the economy returns earlier into the next lockdown, while afterward the trajectories again evolve similarly until the end. Overall, this short period in which policy A recovers considerably stronger is enough such that policy B results in significantly higher economic costs (Table 10).

Our second trade-off, the observation that reducing the lockdown intensity leads to lower economic losses but higher mortality, can not only be derived from the comparison of policies B and C, which both have a threshold of $\beta ^l = 5$, but also for $\beta ^l = 50$ by considering the dotted red curve moving to the upper left from point A in Fig. 6. The dynamics in Fig. 7a illustrate that less reduction in consumption activity (policy C vs. B) leads to more infections and higher mortality. Considering the GDP dynamics (Fig. 7b), one can observe that the stricter lockdown of policy B imposes a strong initial shock on the economy and forces the GDP trajectory on a lower path compared to policy C over the entire course of the simulation. Comparing the slopes of the red and the black line in Fig. 6 clearly shows that the trade-off between reducing economic loss and increasing mortality is less pronounced if the intensity of the lockdown is varied rather than the lockdown threshold. Hence, we obtain the following observation.

#### Result 3

The increase in expected mortality associated with a given decrease in GDP loss is smaller if the reduction in GDP loss is obtained through a reduction in lockdown intensity $\alpha ^l$ compared to an increase in the threshold $\beta ^l$.

Together, Results 1-3 identify a kind of ‘efficiency frontier’ spanned by policies of varying intensity of lockdowns with low threshold (C,B). As one might expect, the frontier is characterized by a trade-off between mortality and economic loss, but policies switching between strict lockdowns and full openings are above the frontier if the threshold for entering a lockdown in such policies is high (A). A policy with a continuous weak lockdown (D), on the other hand, is close to the frontier and dominates policies with high incidence thresholds like A. Whereas so far we have only considered the expected effects of the different policies, in the next result we consider the ex ante uncertainty about mortality and GDP loss, i.e., the variance of these indicators across runs.

#### Result 4

Similar to the trade-offs before, policies differ significantly with respect to the variance of resulting GDP loss and mortality. Effects of policies with a ‘weak lockdown’ (C, D) can be predicted with higher certainty than for policies inducing switches between strong lockdown and full opening (A, B) in terms of GDP loss. In turn, for these policies (C,D) the overall mortality is harder to predict in comparison with policies A and B.

Considering the standard deviation for the different indicators across batch runs, Table 1 shows that the GDP loss induced by policies with weak lockdowns (C,D) varies much less across runs than the GDP loss under policies A and B. Put differently, the economic implications of policies C and D are much better predictable compared to alternative approaches. The downside for this difference is that under policies C and D the variance of the dynamics of infected is substantially larger compared to policies A and B. In particular, for policy A the timing for the second wave is predictable while for policy B, the second wave is prevented by many small lockdowns without varying strongly across runs. In contrast, under policies C and D second large waves (slowly) develop for some of the runs and hence the infection dynamics are harder to predict. Overall, a trade-off in uncertainty emerges with a higher prediction accuracy in GDP loss (C,D) or in mortality (A,B).

### Effects of a variation of the virus infectiousness

Whereas in the previous section we examined the relationship between different lockdown policy designs and the (ex ante) uncertainty of the policy effects with respect to our two key indicators, in this section we focus on the question of how robust our insights about policy effects are with respect to a variation in the infectiousness of the virus and with respect to the absence of the virus mutation. Examining such robustness seems important since in particular at the beginning of the pandemic data about the basic infection probability $p_{inf}$ and the effectiveness of individual protection measures ($\xi$) is hardly available. Hence, one should account for substantial uncertainty about these parameters. Furthermore, the occurrence of (more infectious) mutations can hardly be predicted both with respect to the timing of such an occurrence and the properties of the mutations. Although we do not carry out an extensive exploration of the effects of different pandemic scenarios, we develop some intuition on how strongly the effects of our main policies change under such variations by comparing two alternative scenarios with our default scenario considered so far. First, we consider a counterfactual scenario in which no mutations occur after the initial outbreak of the pandemic, such that throughout the entire considered time horizon of 18 months only the original version of the virus with the calibrated infection probability $p_{inf}$ is active. Compared to our default scenario where a more infectious mutation occurs after several months, this captures a situation with a weaker diffusion of the virus. We refer to it as the *no mutation* scenario. Second, we study a scenario where the value of $p_{inf}$ is increased by 25% compared to our standard calibration and again no mutation occurs. Compared to our default scenario, this corresponds to a situation where initially the infectiousness of the virus is larger, but in the long run it is lower. (The infectiousness of the mutation in our default scenario is 50% above $p_{inf}$.) We refer to this as the *higher* $p_{inf}$ scenario.

**Fig. 8:**
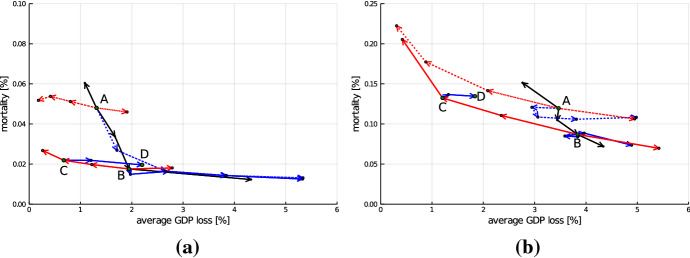
Comparison for scenarios **a** without mutation and **b** with higher $p_{inf}$. All points correspond to averages across the 50 runs. GDP loss [%] on the x-axis measured as loss averaged over simulation time span of 728 periods (24 months) as a percentage of baseline and mortality [%] on the y-axis expressed as a percentage of population

Figure 8 shows the average values of mortality and GDP loss (across batch runs) under the different policies in the two scenarios. Hence, the panels correspond to Fig. 6 for the default scenario. It can be seen that several of the obtained insights remain valid. In particular, both variations of $\alpha ^l$ and $\beta ^l$ are associated with a trade-off between reduction in mortality and GDP loss (Result 2) and the curve associated with a variation of the lockdown threshold $\beta ^l$ (black line) is substantially steeper than the line associated with a variation of the lockdown intensity $\alpha ^l$ (red line), see Result 3. Also, the insight that a policy with a low threshold and weak lockdown (C) dominates a policy with a larger threshold and stronger lockdown (Result 1) carries over to both alternative pandemic scenarios. The comparison between our benchmark policy A and a policy of continuous weak lockdown (D), however, changes if we move from our default scenario to the *no mutation* case. Whereas the mortality is still significantly smaller under policy D compared to A, in the *no mutation* scenario the GDP loss under A is smaller compared to the loss arising under the continuous weak lockdown D. Actually, the GDP loss induced under D hardly differs between the default and the *no mutation* scenario, whereas the loss under policy A is substantially smaller if no mutation occurs. This is not surprising, since under policy D the economic restrictions remain unchanged until all measures are lifted after 18 months, regardless of infection dynamics. Under the benchmark policy A, the duration of the lockdown is much smaller in the *no mutation* scenario than in the default case, since without the occurrence of the mutation the second wave on average occurs later and a third wave is avoided.

**Fig. 9:**
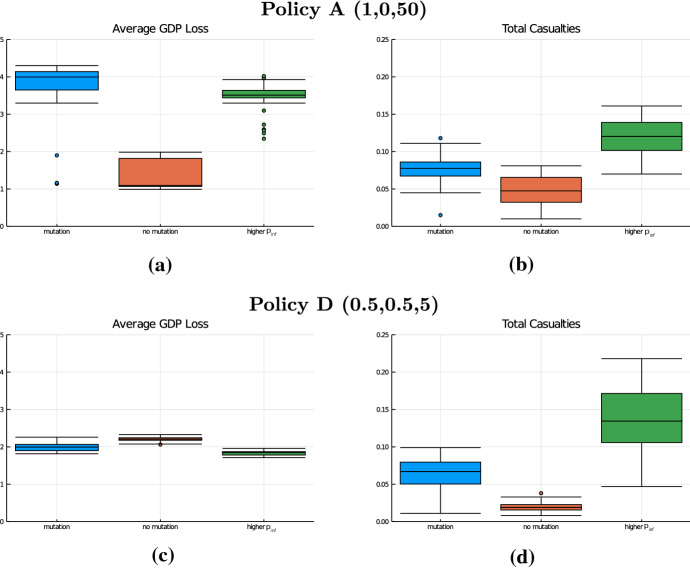
Box plots for the scenarios *mutation* (default, blue), *no mutation* (red) and *higher* $p_{inf}$ (green) over 50 batch runs. **a**, **c** GDP loss [%] measured as loss averaged over simulation time span of 728 periods (24 months) as a percentage of baseline and **b**, **d** mortality [%] expressed as a percentage of population (color figure online)

In Fig. 9, the distribution of outcomes (across the 50 batch runs) under policies A and D are compared across the three considered pandemic scenarios. Comparing panels (a) and (c) illustrates that a continuous weak lockdown policy does not only ensure a low variance of the GDP loss across runs, i.e., good ex ante predictability of the effect on GDP, but also exhibits low sensitivity of the induced GDP loss with respect to a variation in the pandemic scenario. The benchmark policy A induces significantly larger GDP losses compared to C under the default and higher $p_{inf}$ scenarios, but, as discussed above, leads to smaller losses in the no mutation scenario. The variation of mortality across the three scenarios, on the other hand, is similar under both policies.

### Policy phase-out under vaccination rollout

In our main analysis, we assumed that a vaccine is administered instantaneously to the entire population and works with 100% effectiveness. This means the pandemic ends abruptly as soon as the vaccine becomes available. However, in the real world, production, delivery, and administering take a considerable amount of time, a fraction of the population is not willing to participate in vaccination programs and vaccines are not 100% effective. Furthermore, vaccine effectiveness tends to vanish over time. In this subsection, we examine the effects of a constrained vaccination program, where vaccine administration is limited to a certain number of doses per day. A crucial policy question in this setting is to find the optimal stopping point at which virus containment measures should be terminated. For the sake of simplicity, we combine the administration of the first and possible second dose to a single point in time. All parameters of the vaccination program have been set to values, which resembles the situation in Germany. Compared to our default setting vaccinations start at an earlier point in time (day 338), vaccination speed is 0.337% of the population per day and 75% of the population are willing to get vaccinated. This implies that the vaccination rollout is completed on day 561, which corresponds exactly to the day at which in our default model the entire population becomes immune to the virus. According to procedures in most countries, old agents get vaccinated with priority. We assume that vaccine effectiveness declines at a constant rate of 0.34%-points per day, which implies that the effectiveness after 6 months is 23% in line with empirical data (Nordström et al. 2022).

**Fig. 10:**
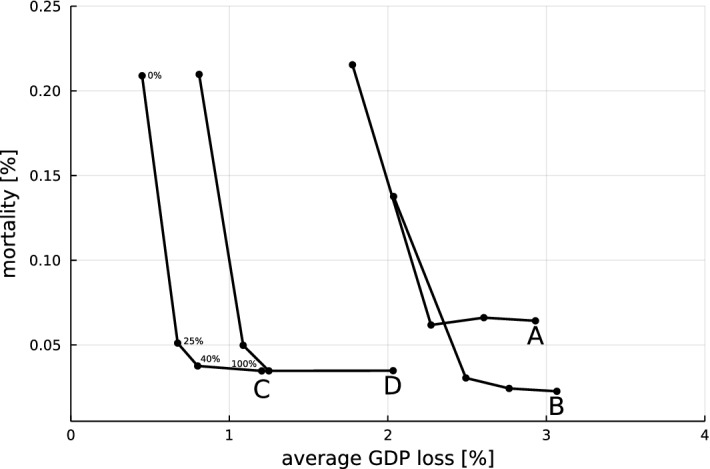
Effect of terminating measures early with vaccination rollout. Lines A-D correspond to the four policy scenarios (Table 2). Measures are terminated after 0, 25, 40 or 100% of the population have been offered a vaccination. For detailed data on GDP loss, mortality, lockdown duration, number of lockdowns and public account deficit, see Appendix C

Figure 10 shows the average GDP loss and mortality under the modified vaccination program for the four policies (A-D) discussed above. Virus containment measures were terminated after 0%, 25%, 40% or 100% of the population had the opportunity to get vaccinated. In the 100% scenario, the administration of the last dose coincides with the vaccination day from our main analysis. Hence, this scenario can be used, to assess the impact of explicitly modeling the vaccine rollout rather than making the stylized assumption that the entire population becomes vaccinated on a certain day, as we did in the previous sections. In all scenarios A-D, both mortality and GDP loss are significantly lower when the vaccine has to be rolled out, compared to our main analysis. The lower mortality can be explained by the fact, that the vaccination program starts earlier with priority for the old age-group. GDP loss is lower, because virus spread is reduced, well before all agents have been vaccinated and the economy therefore spends less time in lockdown. The relationship between the four policy scenarios, however, is qualitatively similar to our main analysis, indicating that our results also apply to a more realistic scenario with a vaccination rollout and decreasing vaccine effectiveness over time. Concerning the optimal point in time for containment measures to be phased out, Fig. 10 shows that in all policy scenarios, mortality is significantly higher, if containment measures are terminated as soon as the vaccination program starts. However, there is no significant difference in mortality between ending the measures after vaccination is complete (100%) and ending the measures once 40% of the population have been offered a vaccination. GDP loss on the other hand is significantly higher in the 100% case, suggesting that virus containment measures should be terminated earlier. This result is driven by two factors: 1) old agents, which have a much higher case mortality rate are given priority and therefore get vaccinated first, 2) virus spread is already noticeably reduced if a fraction of the population is immune. This is particularly helpful in the low-threshold setting (B-D), where infection numbers have been brought down to low values, before lifting the measures.

## Conclusions

In this paper, we develop an agent-based model capable of jointly describing the epidemic and economic effects of measures aimed at containing the COVID-19 pandemic. We show that the calibrated model replicates the economic and epidemic dynamics in Germany in the first 6 months after the COVID-19 outbreak well and employ the model to compare the effects of different alternative policy approaches. Our analysis identifies an efficiency frontier of policies with respect to induced expected virus mortality and GDP loss and shows that policies on that frontier are characterized by small incidence thresholds and that policies with continuous weak lockdowns are close to that frontier as well. Policies characterized by switches between strict lockdowns and full openings based on large incidence thresholds are strictly dominated by frontier policies and also give rise to substantially larger ex ante uncertainty about the actual economic loss to be induced by the policy. We also show that most of these insights are robust with respect to variations of the pandemic scenarios.

Whereas these results have been obtained in calibration of the model based on German data and the COVID-19 pandemic, the mechanisms underlying our findings clearly apply more generally such that these policy insights should carry over to other economies with a similar structure as well as to other pandemics driven by similar kinds of virus transmission.

From a methodological perspective, this approach, which explicitly captures individual interactions related to economic activities, allows us to jointly study the epidemiological and economic effects of different containment measures and to shed light on the interplay between economic activity and propagation of the virus. Due to the flexible microstructure of our model and the explicit representation of virus transmission through interactions between agents, our analysis can be extended in many directions, such as incorporating heterogeneity of infection probabilities across individuals, a social network structure, or different vaccination strategies.

## Data Availability

The model has been implemented in Julia; the code is open source and can be downloaded from GitHub: https://github.com/ETACE/ace_covid19.

## A: Detailed model description

In this appendix, we give a full description of the model and its calibration. Before we describe the economic model (Sect. A.2), we shortly summarize the timing in the model (Sect. A.1). Afterward, we go through the transmission model (Sect. A.3). Finally, we summarize the calibration and initialization including all data sources (Sect. A.4). Some parts appear already in the main text; however, to have a consistent description, we describe all parts of the model in full detail in this section.

### A.1: Timing

The basic unit of time in the model is one day, denoted by $t \in \mathbb {N}_+$. The economic activities of the agents, however, take place on a weekly basis, where firms’ production planning, labor market activities and delivery to the malls all take place at the first day of the week. Households’ consumption is spread out during the week since each household has, for each sector, a (randomly determined) shopping day during the week. In what follows, we denote by $w \in \mathbb {N}_+$ the weeks during the simulation runs and when indexing a variable with the subscript ‘*w*’ we always refer to the first day of week *w*.

### A.2: The economic model

#### Firms

A firm $i \in \textbf{F}_w$ acts as a producer on the goods market and as employer on the labor market. It is assigned to one of the private sectors $k \in \{M,S,F\}$ and delivers to a sector-specific mall that only sells the products of sector *k*. Thus, all firms belonging to the same sector *k* compete on the product market and form a set of direct competitors $\textbf{F}_{k,w}$ of size $n_{k,w}$ in week *w*.

A firm *i* is characterized by a firm-specific level of labor productivity $A_{i}$ that is constant over time. The output of a firm is produced with labor as the only input. Denoting by $L_{i,w}$ the number of workers employed by firm *i* in week *w*, the output of that firm is given by

4 $$\begin{aligned} Q_{i,w} = A_{i} L_{i,w}. \end{aligned}$$

Production takes place on a weekly basis, on the first day of the week. The output is delivered to the mall where each firm keeps an inventory stock. While the inventory is replenished once per week at the day of production, the products in the mall inventory can be sold every day.

The output planning of a firm is based on a simple inventory rule with adaptive demand expectations, where $\hat{D}_{i,w}$ is the expected demand, which is updated according to

5 $$\begin{aligned} \hat{D}_{i,w} = (1-\rho ^D)\hat{D}_{i,w-1} + \rho ^D \; D_{i,w-1}, \end{aligned}$$

where $\rho ^D \in (0,1)$ is a persistence parameter of the expectations and $D_{i,w-1}$ is the sum of the daily sales in the previous production and sales cycle. Denote by $Y_{i,w}$ the inventory stock of firm *i* in the mall at the end of week *w*. The planned output quantity for the current production cycle is then determined by

6 $$\begin{aligned} \tilde{Q}_{i,w} = \left\{ \begin{array}{ll} (1 + \chi _k) \hat{D}_{i,w} - (1 - \delta _k ) Y_{i,w-1}, &{}\text {if }Y_{i,w} > 0\\ (1 + \iota \cdot \chi _k) \hat{D}_{i,w} &{} \, \text {if }Y_{i,w} = 0\\ \end{array} \right. \end{aligned}$$

where $\chi _k>0$ captures the size of a sector-specific inventory buffer and $\iota > 1$ captures that firms might increase their buffer when their stock was sold out in the previous period since they take this as a signal for an expansion in demand. Parameter $\delta _k \in [0,1]$ describes a sector-specific depreciation of the inventory at the end of the sales cycle.

For reasons of simplicity, we abstract from production time and the produced quantity is delivered to the mall at the beginning of the week before consumption starts. The inventory stock then is updated every day depending on the weekly inflow of the replenishment and the daily outflow of sales. At every iteration *t*, the inventory stock in the mall changes according to

7 $$\begin{aligned} Y_{i,t} = \left\{ \begin{array}{ll} (1 - \delta _k ) (Y_{i,t-1} - {X}_{i,t-1}) + Q_{i,t} &{}\text {if }t \ mod \ 7 = 1,\\ Y_{i,t-1} - {X}_{i,t-1} &{} \, \text {else. } \\ \end{array} \right. \end{aligned}$$

Given the planned production volume and firm’s production technology, the labor demand of the firm reads

8 $$\begin{aligned} \tilde{L}_{i,w} = \frac{\tilde{Q}_{i,w}}{A_i}. \end{aligned}$$

Depending on the size of the workforce $L_{i,w-1}$ employed in the previous production cycle, the labor demand $\tilde{L}_{i,w}$ either implies to hire additional workers or to dismiss some redundant workers of the firm. In the former case, i.e., if $\tilde{L}_{i,w} > L_{i,w-1}$, the firm has $L^V_{i,w} = \tilde{L}_{i,w} - L_{i,w-1}$ vacancies from which, depending on the outcome of the labor market, $L^F_{i,w} \le L^V_{i,w}$ will be filled. In the latter case, the firm has $L^R_{i,w} = L_{i,w-1} - \tilde{L}_{i,w}$ redundancies and the firm randomly chooses $L^R_{i,w}$ workers from the set $\textbf{W}^F_{i,w}$ of current employees to be fired. Altogether, the size of the workforce evolves according to

9 $$\begin{aligned} L_{i,w} = \left\{ \begin{array}{ll} L_{i,w-1} + L^F_{i,w} &{}\text {if } \tilde{L}_{i,w} > L_{i,w-1} \\ L_{i,w-1} - L^R_{i,w} &{} \, \text {else.} \\ \end{array} \right. \end{aligned}$$

The weekly wage paid by firms is assumed to be constant over time. It is sector-specific and proportional to the average productivity $\bar{A}_{k}$ of the sector *k* in which firm *i* is active, i.e.,

10 $$\begin{aligned} w_i = w_k = \psi _k \bar{A}_{k} \text { with }\psi _k = \frac{(1-\chi _k \delta _k)}{(1+\lambda _k)(1+\underline{\mu }_k)}>0, \end{aligned}$$

where the sector-specific wage factor $\psi _k$ captures that the expected return from each unit of labor differs between sectors not only due to labor productivity, but also due to differences in expected depreciation of inventory stocks ($\chi _k \delta _k$), the ratio between fixed costs and labor costs ($\lambda _k$) and the mark-up ($\mu _k$).

The firm applies mark-up pricing with an endogenous mark-up $\mu _{i,w}>0$ on unit costs to determine the price of its product. The unit costs of a firm are determined by the variable labor costs and fixed costs $c^F_i$ and are given by

11 $$\begin{aligned} c_i = \frac{w_i + \frac{c^F_i}{L_{i,w}} }{A_i (1- \chi _k\delta _k)} . \end{aligned}$$

The resulting price of a firm is given by

12 $$\begin{aligned} P_{i,w} = (1+\mu _{i,w}) c_i. \end{aligned}$$

The mark-up is updated at the day of production and depends on the market share of the firm. Denote by $$s_{i,w} $$ the market share (in terms of sold quantity) of firm *i* on its relevant market in week *w*, then the mark-up equals

13 $$\begin{aligned} \mu _{i,w+1} = \underline{\mu }_{k} + s_{i,w} \cdot (\bar{\mu }_k - \underline{\mu }_k), \end{aligned}$$

where $\bar{\mu }_k$ and $\underline{\mu }_k$ are parameters determining the upper and, respectively, lower bound for the mark-up in sector *k*.

Accounting takes place at the day of production and is related to the previous production cycle. The profits of firm *i* accounted for in period *w* read

14 $$\begin{aligned} \Pi _{i,w} = P_{i,w}{D}_{i,t} -L_{i,w} w_i -c_i^F. \end{aligned}$$

Profits are negative if revenues are not sufficiently large to cover the wage bill plus the fixed costs $c_i^F$. The liquidity of the firm evolves according to

15 $$\begin{aligned} S_{i,w} = S_{i,w-1} + \Pi _{i,w-1} - \max [0,\tau _{w-1} \Pi _{i,w-1}] - d_{i,w} \end{aligned}$$

Here, $\tau _w$ is the tax rate for corporate taxes on (positive) profits and $d_{i,w}$ are dividends paid out to the firm’s shareholders. For the dividends, we define a dividend rate $\zeta \in (0,1)$ and a threshold level of liquidity being proportional to the average firm revenues over the last *T* weeks, i.e.,

16 $$\begin{aligned} \tilde{S}_{i,w} = \varphi _k \ \frac{1}{T}\sum _{\tau =0}^{T-1} P_{i,w-\tau } D_{i,w-\tau }. \end{aligned}$$

Firms pay out their entire (after tax) profits as dividend once their liquidity is above that threshold, otherwise they save a fraction of the profits thereby increasing their liquidity:

17 $$\begin{aligned} d_{i,w} = \left\{ \begin{array}{ll} (1-\tau _w) \max [0, \Pi _{i,w}] &{}\text {if }S_{i,w-1} + (1 - \tau _w) \max [0, \Pi _{i,w}] >\tilde{S}_{i,w} \\ \zeta (1-\tau _w) \max [0, \Pi _{i,w}] &{} \, \text {else,} \\ \end{array} \right. \end{aligned}$$

with $\zeta < 1$. The dividends as well as fixed costs are distributed equally to all households.

If a firm has negative liquidity after accounting, it has to declare bankruptcy. In this case, the firm becomes inactive and has to dismiss all workers. At the same time, the firm’s inventory stock is fully written off.

#### Households

The economy is populated by $m_t$ households. A household $h \in \textbf{H}_t$ acts as customer on the goods market and, depending on her age, as an employee on the labor market.

Households are divided into a young cohort $\textbf{H}_t^Y$ and an old cohort $\textbf{H}_t^O$. Members of the old cohort are retired, whereas households in the young cohort constitute the labor force of the economy. A young household can be employed or unemployed. If a household is unemployed, she enters the labor market to search for a new job.

Households have work-related skills that can only be utilized in one of the sectors $k \in \mathbf \{M,S,F\}$ and cannot be transferred to other sectors. Thus, households are uniquely assigned to one sector and constitute the sector-specific labor supply $\textbf{L}^S_{k,t}$. Apart from the private sectors, there is also a public sector (indexed by $k=P$) that does not produce any market goods. In this sector, the government operates $n_P$ offices and households that work for the government as civil servants have a permanent and secure job.

We assume that, in each sector, there is a fixed proportion $h^{WFH}_k$ of workers working on tasks that can potentially be done from home. The set composed of these workers is denoted by $\textbf{L}^{WFH}_{k,t} \subset \textbf{L}^S_{k,t}$.

Depending on their age and employment status, households have different income sources. Employed households earn a labor income $\omega _{h,w}$ equal to the wage $w_k$ of the sector *k* in which a household is employed. Unemployed households, instead, receive unemployment benefits $u_{h,w}$ from the government that correspond to a fraction $\nu$ of their last labor income. Old households live on pensions of level $w^P$ that are paid by the government and are uniform and constant over time for all retirees in the economy. Additionally, all households receive a capital income that correspond to an equal share of the fixed costs paid by firms and dividends distributed by the firms, i.e.,

18 $$\begin{aligned} I_{h,w}^{Cap} = \frac{1}{m_w} \sum _{\forall i \in \textbf{F}_{w}} (d_{i,w} + c_i^F). \end{aligned}$$

Altogether, the overall gross income $I_{h,w}$ of household *h* in week *w* equals

19 $$\begin{aligned} I_{h,w} = \left\{ \begin{array}{ll} \omega _{h,w} + I_{h,w}^{Cap} &{} \text {if employed}, \\ u_{h,w} + I_{h,w}^{Cap} &{} \text {if unemployed}, \\ w^P + I_{h,w}^{Cap} &{} \text {if retired.} \\ \end{array} \right. \end{aligned}$$

All sources of income are subject to income tax. Given tax rate $\tau _w$, the net income of household *h* is then

20 $$\begin{aligned} {I}^N_{h,w} = (1-\tau _w) I_{h,w}. \end{aligned}$$

On the first day of the week, the household decides on the budget $C_{h,w}$ that she plans to spend in the coming week. For the consumption and saving decision, the household takes into account an average net income

21 $$\begin{aligned} \bar{I}^N_{h,w} = (1-\rho ^I) \bar{I}_{h,w-1} + \rho ^I I^N_{h,w} \end{aligned}$$

as well as her total wealth $W_{h,w}$, which consists of her money holdings. The notional consumption budget is determined according to the consumption rule

22 $$\begin{aligned} C_{h,w}= \bar{I}^N_{h,w} +\kappa \cdot (W_{h,w}-\Phi \cdot \bar{I}^N_{h,w} ), \end{aligned}$$

where the parameter $\Phi$ is the target wealth/income ratio. This formulation is motivated by the ‘buffer stock’ theory of consumption, which is backed up by theoretical arguments and substantial empirical evidence (Deaton 1991; Carroll and Summers 1991). The parameter $\Phi$ describes how large the targeted buffer is relative to income, and $\kappa$ indicates how sensitively consumption reacts to deviations of the actual wealth-to-income ratio to the target level.

Finally, the consumption budget $C_{h,w}$ is allocated to the different sectors. In principle, the budget that a household *h* tries to spend for products from sector $k \in \{M,S,F \}$ is determined by a fixed allocation across sectors, i.e.,

23 $$\begin{aligned} \tilde{C}^S_{h,k,w}= c_k \; C_{h,w}. \end{aligned}$$

However, sector $k=F$ is different from the other sectors in a way that it includes essential goods implying that households try to avoid large spending cuts for these products. Hence, the actual consumption budget allocated to the essential sector *F* is

24 $$\begin{aligned} {C}^S_{h,F,w}= \max \left[ c_{F} \; C_{h,w}, \min \left[ (1-\phi ){C}^S_{h,F,w-1} , C_{h,w}\right] \right] . \end{aligned}$$

The remaining budget, instead, is distributed proportionally among the non-essential sectors $k\ne F$ according to the consumption quotas $c_k$ such that

25 $$\begin{aligned} {C}^S_{h,k,w}= \frac{c_k}{\sum _{l\in \textbf{K} \setminus \{k^*\}} c_l } ( C_{h,w} - {C}^S_{h,F,w} ). \end{aligned}$$

The actual expenditure in a certain sector on the households sector-specific shopping day can deviate from planned ones due to rationing (see below). Denote by $E_{h,t}\ge 0$ the total expenditures for consumption on day *t*, then the savings of household *h* evolve according to

26 $$\begin{aligned} W_{h,t}= \left\{ \begin{array}{ll} W_{h,t-1} - E_{h,t-1} + I^N_{h,t} &{}\text {if } t \ mod \ 7 = 1\\ W_{h,t-1} - E_{h,t-1} &{} \, \text {else. } \\ \end{array} \right. \end{aligned}$$

#### Labor market interactions

The labor market is modeled as a decentralized market with separated sub markets for each sector. The labor market operates every first day of the week to match open vacancies and job seekers. All firms belonging to sector *k* that have open vacancies $$ L^V_{i,w}>0$$ try to get matched with the unemployed workers $$\textbf{U}^S_{k,w}$$ searching for a job in sector *k*. All households $$h \in \textbf{W}^S_{P,w}$$ that work in the public sector stay with their employee throughout the simulation run and are never active on the labor market.

The matching process is modeled in a way that firms open vacancies in random sequence and unemployed job seekers with appropriate skills apply. The firm then hires on a first-come-first-serve basis. If at the time of the announcement of the job opening there are no unemployed job seekers with appropriate skills, the firm is rationed and can only hire again in the following week.

More precisely, suppose $\textbf{V}_{k,w}$ is the shuffled set of firms in the queue of sector *k* in week *w* and $v_l \in \textbf{V}_{k,w}$ is the firm ranked at the *l*th position. Denote by ${\tilde{L}}^S_{k,w,l}$ the number of unemployed in sector *k* after firm $v_l$ has been active on the labor market with $\tilde{L}^S_{k,w,0} = |\textbf{U}^S_{k,w}|$. Then, for all firms $v_l \in \textbf{V}_{k,w}$, the number of hired, respectively, fired workers in week *w* is given by

27 $$\begin{aligned} \begin{array}{ll} L_{i,w}^F = \min [{\tilde{L}}_{i,w} - L_{i,w-1}, {\tilde{L}}^S_{k,w,l-1}] &{} \text{ if } {\tilde{L}}_{i,w} \ge L_{i,w-1} \\ [.2cm] L_{i,w}^R = {\tilde{L}}_{i,w-1} - L_{i,w} &{} \text{ else }. \end{array} \end{aligned}$$

The number of unemployed evolves according to

$$\begin{aligned}{\tilde{L}}^S_{k,w,l} = {\tilde{L}}^S_{k,w,l-1} - L_{i,w}^F + L_{i,w}^R.\end{aligned}$$

Hence, a firm might be rationed on the labor market if the number of job seekers at the time the firm is active on the market is below its labor demand.

#### Goods market interactions

Once per week, a household randomly determines a shopping day for each sector within the next 7 days. At the respective shopping day for sector *k*, the household *h* visits a mall in which those products are sold. Denote by $\textbf{C}_{k,t}$ the sorted set of costumers shopping in sector *k* at day *t* and by $c_{l} \in \textbf{C}_{k,t}$ the consumer at the *l*th position in the queue. Furthermore, denote by ${\tilde{Y}}_{i,t,l}$ the inventory of firm *i* in the mall after consumer $c_l$ has completed her shopping and by $\textbf{A}_{t,l}$ the set of active firms at that point, i.e., those firms *i* for which ${\tilde{Y}}_{i,t,l} > 0$ holds.

Consumer $c_l$ draws a random subset ${\Omega }_{c_l,k,t} \subseteq \textbf{A}_{t,l}$ of size $\eta$ of the products offered by active firms in the mall. The decision which product $i \in {\Omega }_{c_l,k,t}$ of sector *k* to purchase is based on a logit choice model. The probability to buy the product from firm *i* offered at price $P_{i,w_t}$ is

28 $$\begin{aligned} \mathbb {P}[c_l \text { selects } i \in {\Omega }_{c_l,k,t} ] = \frac{\exp ( - \gamma ^C \log (P_{i,w_t}))}{ \sum _{\forall j \in \Omega _{c_l,k,t}} \exp ( - \gamma ^C \log (P_{j,w_t} ))}, \end{aligned}$$

where $\gamma ^C$ is a parameter for the price sensitivity of households and $w_t$ denotes the week of day *t*. The notional quantity to purchase is then

29 $$\begin{aligned} \mathcal {C}_{c_l,i,t} = \min \left[ \frac{C^S_{c_l,k,w_t}}{P_{i,w_t}},{\tilde{Y}}_{i,t,l-1} \right] . \end{aligned}$$

The stock of firm *i*’s goods still available at the mall is updated according to

30 $$\begin{aligned} {\tilde{Y}}_{i,t,l} = {\tilde{Y}}_{i,t,l-1} - \mathcal {C}_{c_l,i,t}. \end{aligned}$$

If ${\tilde{Y}}_{i,t,l} = 0$, the firm becomes inactive in the mall at this point and only becomes active again at the first day of the following week when new goods are delivered to the mall. If there are no active firms in the mall when a household *h* visits the mall or if the chosen firm is not able to supply to total amount demanded, i.e., ${\tilde{Y}}_{i,t,l-1} < \frac{C^S_{c_l,k,w_t}}{P_{i,w_t}}$, the consumer is rationed and returns to the mall again the following day. Unspent consumption budget for sector *k* is added to the household’s savings.

#### Government and public sector

The government collects income and profit taxes to pay for the wages of civil servants working in one of the $n_P$ offices in $\textbf{G}$, unemployment benefits and pensions. Additionally, the government can pay subsidies or other financial support to households and firms as part of policies.

Each public sector office $g \in \textbf{G}$ employs a set of civil servants $\textbf{W}^G_{g} \subset \textbf{H}_{0}^Y$ that only changes over time if an employee dies. The total number of civil servants in the economy in week *w* is denoted by $L^P_w$.

Unemployment benefits are based on the last wage of an unemployed households with replacement rate $\nu$. Pensions are uniform for all old households and are a percentage *pen* of the average wage in the economy. Households employed in the public sector earn a wage $w^P$.

Tax collection and distribution of unemployment benefits and pensions takes place at the first day of the week. The tax revenue of the government is the sum of the corporate tax revenues

31 $$\begin{aligned} T^C_w = \sum _{i \in \mathbf {F_w}} \max [0, \tau _w \; \Pi _{i,w}] \end{aligned}$$

and the income tax revenues are

32 $$\begin{aligned} T^I_w = \tau _w \sum _{h \in \textbf{W}_w} \;\omega _{h,w}+ \tau _w\sum _{h\in \textbf{H}_w} I_{h,w}^{Cap} + \tau _w \sum _{h \in \textbf{U}_w} u_{h,w} + \tau _w w^P \; |\textbf{H}^O_w| , \end{aligned}$$

where $\textbf{W}_w$ denotes the set of all employed households in the economy in week *w*. Denoting by $\textbf{U}_w$ the set of unemployed workers in the economy, the public account of the government evolves according to

33 $$\begin{aligned} S^G_w = S^G_{w-1} + T^C + T^I - \sum _{h \in \textbf{U}_w} u_{h,w} - w^P \; |\textbf{H}^O_w| - w_0^S \;L^P \end{aligned}$$

The government adjusts the tax rate over time in order to keep a target level of the public account. In the baseline setup, the tax rate $\tau _w$ evolves according to

34 $$\begin{aligned} \tau _w = (1-\rho ^T)\tau _{w-1} + \rho ^T \;\hat{\tau }_w, \end{aligned}$$

where $\hat{\tau }_w$ is the tax rate that would be sufficient to balance the budget on a target public account level. In particular,

35 $$\begin{aligned} \hat{\tau }_w = \max \left[ 0, \frac{\sum _{h \in \textbf{U}_w} u_{h,w} + w^P \; |\textbf{H}^O_w| + w_0^S \;L^P - \theta S^G_{w}}{\frac{T^C}{\tau } +\sum _{h \in \textbf{W}_w} \omega _{h,w} } \right] , \end{aligned}$$

Note that the target level of public account and the speed of tax rate adjustment might change as part of a policy.

Finally, the government computes the gross domestic product for the last week according to

36 $$\begin{aligned} GDP_w = w_0^S L^P + \sum _{k \in \textbf{K}} \sum _{i \in \textbf{F}_{k,w}} P_{i,w} Q_{i,w}. \end{aligned}$$

### A.3: Virus transmission

#### Social interactions

Social interactions take place on three different occasions (see Fig. 2 for a visual summary). The first type of social interactions occurs at work. Firms and public offices represent the work environment where social contacts in the professional context occur. Suppose $\textbf{X}_{h,t} = \textbf{W}^F_{i,t} \setminus \{h\}$ is the set of household *h*’s colleagues at time *t* (or $\textbf{X}_{h,t} = \textbf{W}^G_{g} \setminus \{h\}$ for public servants). As long as she is not in the short-term program or working from home, the worker faces several potential meetings with her co-workers, where

37 $$\begin{aligned} \tilde{\textbf{X}}_{h,t} = \left\{ \begin{array}{ll} \emptyset &{} h \in (\textbf{W}^{WFH}_{i,t} \cup \textbf{W}^{ST}_{i,t}), \\ \textbf{X}_{h,t} \setminus (\textbf{W}^{WFH}_{i,t} \cup \textbf{W}^{ST}_{i,t}) &{} \text {else,} \\ \end{array} \right. \end{aligned}$$

is the set of co-workers worker *h* can potentially meet during a workday. As defined above, $\textbf{W}^{WFH}_{i,t}$ is the set of workers working from home and $\textbf{W}^{ST}_{i,t}$ is the set of workers on short-time work on day *t*. The realized number of meetings is drawn from a distribution where the maximum contact threshold $n^{w}_k$ might differ across sectors. The number of colleagues $N^{w}_{h,t}$ met by agent *h* is drawn from a uniform distribution with bounds $[0,n^{w}_k]$. The set of actually met co-workers of agent *h* at time *t* is then determined as a random subsample $\textbf{CW}_{h,t} \subset \tilde{\textbf{X}}_{h,t}$ of size $N^{w}_{h,t}$.

The second possibility to interact with other households takes place during shopping. Households visit different shopping malls within a week in order to purchase or consume goods offered by the three private sectors. The maximum number of possible meetings at one shopping day is drawn from a distribution where the upper threshold $n_k^{c}$ is sector-specific. The actual number of people met in the specific mall is given by the fraction of the population going to that mall multiplied by the maximum number of possible meetings across the week. Thus, if one-seventh of the local population is going to that mall, the number of contacts when shopping will be equal to the maximum number of possible contacts. Formally

38 $$\begin{aligned} N_{h,k,t}^{c} = \bar{N}_{h,k,t}^{c} \cdot \frac{\vert \textbf{C}_{k,t} \vert }{\vert \textbf{H}_{t}\vert } \cdot 7, \end{aligned}$$

where $\vert \textbf{C}_{k,t} \vert$ is the number of customers of sector *k* at time *t* and $\textbf{C}_{k,t}=\sum _{i} \textbf{C}_{i,t}$, $\vert \textbf{H}_{t}\vert$ is the number of households at time *t*. $\bar{N}_{h,k,t}^{c}$ is the upper bound number of co-shoppers met by agent *h* in sector *k* at time *t* and is drawn from a uniform distribution with bounds $[0, n_k^{c}]$. We denote by $\textbf{CS}_{h,k,t} \subset \textbf{C}_{k,t}$ the actual set of individuals met while shopping at the local mall, which is drawn as a subsample of size $N_{h,k,t}^{cs}$. Multiple meetings with the same household are possible.

Finally, households engage in other social activities, where the frequency of social interactions differs for meetings between different age-groups. In particular, the number of contacts for each type of meeting is drawn from a uniform distribution whose upper bound $n_{a,a}^{p}$ with $a \in \{y,o\}$ reflects the interaction patterns between members of different or the same age-group. In case of a positive number of contacts for period *t*, potential partners are drawn among the population belonging to the specific age-group. $\textbf{H}^{a}_{-h,t}$ is the set of households belonging to a specific age excluding agent *h* at time *t*. We select the number of people $N^{a,a}_{h,t}$ met by agent *h* at time *t* by drawing from a uniform distribution with bounds $[0,n_{a,a}^{p}]$ and draw the set of actual contacts $\textbf{SA}_{h,t}^{a} \subset \textbf{H}^{a}_{-h,t}$ from a specific age-group met by agent *h* at time *t* as a random subsample of size $N^{a,a}_{h,t}$.

#### Pandemic dynamics

Households differ with respect to their health states. At every instant of time *t*, each household *h* may be in one of four states. ‘Susceptible,’ not yet been exposed to the virus and thus not immune, ‘Infected’ already contracted the virus, ‘Recovered’ have been infected, survived the virus and acquired immunity and ‘Deceased’ died from the virus. We further subdivide the infected state into three different phases, which do matter in terms of virus transmission: We distinguish between a latency phase of length $t_{lnt}$, an infectious phase of length $t_{inf}$ and a post-infectious period where one has not yet recovered. $\bar{t}_{rec}$ is the maximum number of days being infected or the recovery time. The set of households belonging to the four health states are denoted by $\textbf{S}_t$, $\textbf{I}_t$, $\textbf{R}_t$ and $\textbf{D}_t$, respectively. The set of infectious agents is denoted by $\textbf{I}^{inf}_t \subseteq \textbf{I}_t$ and that of newly infected people is denoted by $\textbf{T}_t$. Thus the population of alive households evolves together with the epidemic and changes over time such that:

39 $$\begin{aligned} \textbf{H}_t = \textbf{S}_t + \textbf{I}_t + \textbf{R}_t. \end{aligned}$$

In other words, the population decreases due to death from the disease while we abstract from other demographic dynamics such as births and other causes of death. We assume that the initial stocks of infected, recovered and deceased individuals are set equal to zero. Hence, before the outbreak of the epidemic, the entire initial population of household belongs to the susceptible group.

At period $t=t_0$, the epidemic starts. The initial infected agents are randomly selected, their state is updated and their recovery countdown starts. The rest of the population stays susceptible and is exposed via three channels through which the infection can be transmitted, social contacts at work, during consumption and other social occasions, where only meetings with infectious households might result in the contagious.

At every contact between an infectious household $h \in \textbf{I}^{inf}_t$ and a susceptible household ${\tilde{h}} \in \textbf{S}_t$ the virus is transmitted with a probability $(1-\xi ) p_{inf}$, where $\xi = 0$ in the absence of any policy measures. An infected agent *h* at each possible day has a small probability $q_{t}^{a}$ to die from the virus. In this case, she is removed from the unemployment list or from her employers list of workers. The number of casualties is updated accordingly. After $\bar{t}_{rec}$ days of infection the household recovers and gains immunity to the virus.

The case fatality rates $q_t^{a}$ with $a=\{y,o\}$ do not only depend on the age of the household, but also on the degree of utilization of intensive care units in the economy at time *t*. In case of an over-utilization, the rate is increasing with $\vert \textbf{I}_t \vert$. In particular, depending on the degree of over-utilization, the age-specific fatality rate is a weighted average between a regular fatality rate $\bar{q}_l^{a}$ achieved with under-utilized intensive care units and a fatality rate $\bar{q}_h^{a}$ that would be achieved if no intensive care capacities would be available. Formally

40 $$\begin{aligned} q_t^{a} = \left[ \frac{\min (n^{icu},u^{icu} \cdot \vert \textbf{I}_t\vert )}{u^{icu} \cdot \vert \textbf{I}_t\vert }\right] \bar{q}_l^{a} + \left[ 1-\frac{\min (n^{icu},u^{icu} \cdot \vert \textbf{I}_t\vert )}{u^{icu} \cdot \vert \textbf{I}_t\vert }\right] \bar{q}_h^{a} \end{aligned}$$

where $n^{icu}$, $u^{icu}$, $\vert \textbf{I}_t\vert$ are, respectively, the number of intensive care beds available, the fraction of infected individuals in need of intensive care and the total number of actual infected.

We assume that after $t_{vac}$ days from the beginning of the pandemic, a vaccine becomes available on the market and agents start to get vaccinated. For most of our analysis, we assume that all susceptible households are assumed to receive vaccination immediately and the epidemic ends as soon as the remaining infected households recover or die. For a more sophisticated analysis, we also consider the case, where the vaccine can only be administered at a finite vaccination speed ($s_{vac}$), there is an age-group-specific willingness to become vaccinated ($will_{vac}^o$, $will_{vac}^y$), vaccines only work with a certain effectiveness $eff_{vac}$ and agents individual vaccine effectiveness may decrease at a daily rate $eff_{vac}^{dec}$ after administration.

### A.4: Calibration

#### Economic activities

Following German demographic data we set the fraction of young households to $a^Y_0 = 75\%$ capturing that the number of individuals belonging to the age-group between 18 and 65 years in the German population is about three times the number of individuals with an age above 65 years (Statista 2019a, b).

The productivity level of a firm *i* in sector *k* is a random variable following a uniform distribution from an interval around a sector-specific average productivity $\bar{A}_k$ (Statistisches Bundesamt (Destatis) 2020b). Sectoral wages are proportional to the average productivity in the sector and their level is chosen such that the average price is equal to one, taking into account (average) firm mark-ups and fixed costs in each sector (given the firm’s mark-up). Productivity and wages are measured in units of 1.000/52 €, such that a weakly output of 1 unit corresponds to an annual GDP of 1.000 €. The parameters determining the allocation of households consumption expenditures across the three private sectors, $c_M=21\%$ $c_S=50\%$ $c_F=29\%$, are based on Statistisches Bundesamt (2017). The labor supply in the three private sectors manufacturing, service and food, i.e., the fraction of the labor force with the corresponding skills, are calculated based on the allocation of consumption expenditures across the three sectors and the average labor productivity in a way to generate comparable unemployment rates across sectors. This gives fractions $e_M= 11.70\%$, $e_S = 43.62\%$ and $e_F=32.68\%$. The initial number of workers in sector *k* is $m_0 \;\cdot e_k$ and the initial number of firms or, respectively, offices equals $n_k = e_k \; n_0$. The properties of the sectoral structure are summarized in Table 2.

Additional economic parameters, like the firms’ sector-specific inventory buffers or mark-up ranges, are calibrated to generate a stationary GDP per capita and unemployment rate that reasonably match the German economy before the pandemic. In particular, the model generates an annual GDP per capita of 43.013 euro and an unemployment rate of $3.98\%$ for the pre-pandemic period (average over 50 runs), compared to an annual GDP per capita (Eurostat 2020a) of 41.350 euro and an average unemployment rate (Eurostat 2020b) of $3.2\%$ in 2019.

**Table 2:** Sectoral distribution of economic values

	Manufacturing	Service	Food	Public
Workers with sector-specific skills	$11.70\%$	$43.62\%$	$32.68\%$	$12.00\%$
Av. productivity	97	62	48	62
Productivity range	87.3–106.7	58.9–65.1	43.2–52.8	62
Av. wage	76.5	50.1	38.8	59.2
Consumption shares	$21\%$	$50\%$	$29\%$	–

*Notes*: Productivity level of a firm *i* in sector *k* is a random variable following a uniform distribution from an interval around a sector-specific average productivity $\bar{A}_k$ based on German data (Statistisches Bundesamt (Destatis) 2020b). Sectoral wages are proportional to average productivity in sector and their level is chosen such that average price, taking into account (average) firm mark-ups and fixed costs in each sector (given the firm’s mark-up) is equal to one. Productivity and wages are measured in units of 1.000/52 euro, such that a weakly output of 1 unit corresponds to an annual GDP of 1.000 euro. The parameters determining allocation of agent’s consumption expenditures across three private sectors as well as employment share of public sector are based on German data (Statistisches Bundesamt (Destatis) 2020a; Grimault 2020). Labor supply in three private sectors manufacturing, service and food, i.e., the fraction of labor force with corresponding skills, is given by estimated employment shares. These shares are calculated based on allocation of consumption expenditures across the three sectors and average labor productivity

#### Social interaction

Social interactions between households take place at three different occasions, which we calibrate with data reported in a survey on social contacts by Mossong et al. (2008). The actual number of contacts for an agent is a random draw from a uniform distribution between zero and a case-specific upper bound. The first one describes work-related contacts capturing that an employed agent meets co-workers. We assume that an employee meets on average four co-workers during one working day (given an interval with upper bound $n^{w}_k=8$). The second occasion are social contacts that occur during shopping. For the service sector, this for example includes contacts during the visits of a restaurant or a fitness studio. The total number of shopping contacts of a households per day is sector-specific ${n}_{M}^{c} = 10$, ${n}_{S}^{c} = 28$, and ${n}_{F}^{c} =10$, such that the number of potential meetings during consumption of services is considerably higher compared to the other types of goods. Finally, we model other social contacts that happen for example during leisure time. Here, we make a distinction in the frequency of social interaction between age-groups. The actual number of social interactions per day across different age-groups is limited by the upper bounds $n^{p}_{yy} =5$, $n^{p}_{oo} =2$, $n^{p}_{yo} =2$, and $n^{p}_{oy} =4$. This reflects that agents meet within their age-group more frequently.

#### Virus transmission

Our model is calibrated to replicate the first 6 months of the pandemic of the SARS-CoV-2 virus in Germany. Since the pandemic is still ongoing, there is a considerable uncertainty around key parameters of the virus. Our choice of parameters is consistent with the current data on COVID-19. The initial fraction of population infected is based on reported number of infected in Germany on March 9, 2020. Since not all patients infected with SARS-CoV-2 show symptoms, the estimated number of infected individuals differs substantially from the detected number of cases (Bommer and Vollmer 2020). We use the empirical infection and fatality rates (Verity et al. 2020) to estimate a detection rate in Germany. We use their result that 15% of infected are reported to link the number of infected in our model to data giving the reported number of infections. Taking this into account and scaling the number of reported infected in Germany on March 9, 2020, to our population size of 100.000 households yield an initial number of 8 young and 3 old infected households in our model.

The actual value of $p_{inf}$, the probability to be infected when meeting a contagious individual, is unknown in the literature. Instead, we calibrate this value such that in a scenario without any virus containment measures the average reproduction number in initial periods before herd immunity starts to play a role matches the value of $R_0 \approx 2.5$ and hence lies well within the standard range of values reported for this number (Read et al. 2020). Upon infection and after a latency period of five days ($t_{lnt}=5$), agents are infectious for five days ($t_{inf}$=5) (World Health Organisation 2020).

In case a household is infected, she takes $\bar{t}_{rec} = 21$ days to recover. During this time, the household might pass away. The calibration of the individual case fatality rate for the case of not fully utilized intensive care capacities relies on age-structured German data of casualties and reported infected as of the beginning of June 2020, where the total number of reported infected has been allocated to different age-groups (Robert Koch Institut 2020). In that case, the fatality rate for individuals below 65 years is $0.66\%$ of reported infected. For individuals older than 65 years, this rate is $16\%$. Taking into account that only 15 percent of infected are reported, we obtain $q_l^y = 0.099\%$ and $q_l^o=2.4\%$. To capture the effect of a collapsing health system, we extrapolate Italian data collected during periods of over-utilization of local intensive care capacities (Statista 2020a). In case of a congestion of intensive care capacities, we use $q_h^y = 0.27\%$ for young and $q_h^o=7.5\%$ for old patients.

An infected household needs intensive care in $8.5\%$ of the reported cases (Anesi 2020). The assumed number of intensive care beds is 30 per 100.000 households, which is based on German data (Rhodes et al. 2012). Finally, we assume that a vaccine will be available 18 months after the initial spread of the virus. The pandemic related data for our calibration is summarized in Table 3.

**Table 3:** Parameter values related to COVID-19

Parameter from literature	
Recovery period	21 days
Infectious period	5 days
Latency period	5 days
Detection rate	0.15
Reported infections in need of intensive care	$8.5\%$
Intensive care units (ICU)	30 per 100.000
Fatality rates	
Below ICU capacity	
Young	$0.099\%$
Old	$2.4 \%$
Without ICU treatment	
Young	$0.27\%$
Old	$7.5\%$

We use estimation from World Health Organisation (2020) for the recovery, infectious and latency period. To adjust for infected, but undetected cases, we use the estimated rate of asymptomatic cases (Subramanian et al. 2021). The percentage of infected in need of intensive care is an estimation from Rhodes et al. (2012). The actual number of intensive care units (ICU) is taken from German data (Anesi 2020) and scaled to our population size. To get estimates for fatality rates in case the ICU capacity does not exceed its capacity, we use data from the German Robert Koch Institut (2020). Italian data (Statista 2020a) is used for patients without ICU treatment

#### Virus mutation

In September 2020, a mutation of the SARS-CoV-2 was detected in Great Britain (Chand et al. 2020) and has been found in other countries subsequently. This mutation quickly became the most common variant in the UK and accounted for almost two third of new cases in London by mid-December 2020 (Kirby 2021). Other virus mutations have emerged in other areas. With a higher infection probability, the mutation poses new challenges for health authorities. We introduce mutations in our model in the following way.

At a specific day ($t^{mut}$ = 162), a number of agents ($n^{mut}$ = 5) is infected by the mutation. This separates the set of infected agents $\textbf{I}_t$ into two types of infected status, either with the original or the mutated virus $\textbf{I}_t^{mut}$. We rely on British data to calibrate the mutation (Chand et al. 2020) and assume the transmission probability increases by $50\%$ for a household infected with the mutated virus. Hence, in case one of the newly infected agents meets a susceptible household, the infection probability is given by $p_{inf}^{mut} = 1.5 \ p_{inf}$. A household inherits the type from the infecting agent. Finally, we assume that data such as latency period or fatality rate as given in Table 3 is the same for a household infected by the mutation.

#### Policy measures

A whole set of measures has been introduced in many countries, for example in Germany in the beginning of March 2020. These measures include individual prevention measures, working from home where possible, a regulation banning gatherings between more than two people in public spaces (with the exception of families), the closure of a large fraction of stores (apart from supermarkets, and stores for food and other essential products) as well as all hotels and restaurants. In the framework of our model we put all these measures together to a single lockdown policy accompanied with a phase-in period after the implementation of the policy during which the model parameters adjust to their new values.

More detailed, the lockdown is introduced 2 weeks after the first agents are infected. Following empirical data, sector-specific working from home is introduced in manufacturing, service and public sector ($h^{ho}_M = 45\%$, $h^{ho}_S = 30\%$, $h^{ho}_P = 75\%$ of employees), but not in the food sector (Fadinger and Schymik 2020; Möhring et al. 2020). When the working from home measure is active the sector-specific upper bounds for the number of contacts at the workplace (for those not working at home) are reduced to ${n}_{M}^{w} = 4$, ${n}_{S}^{w} = 5$, ${n}_{F}^{w} =8$, ${n}_{P}^{w} =2$. Furthermore, we assume that working from home does not decrease the firm’s productivity level $A_i$. Based on survey data (Lehrer et al. 2020), we assume that, when social distancing is active, the upper bounds of social contacts are reduced to $n^{p}_{yy} =2$, $n^{p}_{oo} =1$, $n^{p}_{yo} =1$, and $n^{p}_{oy} =1$. Finally, when a lockdown is in place the upper bound of the number of contacts during each shopping trip are reduced to ${n}_{M}^{c} = 5$ and ${n}_{S}^{c} = 20$. The reduction in a household’s weekly probability to carry out her activity in manufacturing and services are estimated as $\Delta p^{s,l}_M = 0.1$ and $\Delta p^{s,l}_S = 0.33$. These numbers are based on data on sector-specific reduction in consumption and GDP loss in Germany during the lockdown in March 2020, see (Statista 2020b), and our convention that the consumption reduction during that lockdown corresponds to an intensity of $\alpha ^l = 1$.

With respect to the short-time work scheme, mirroring measures introduced in Germany, we set the ratio of short-time wage and regular wage to $\varphi = 0.7$. Furthermore, the probability that a worker not needed under the current production plan enters short-time work is calibrated to $q^{st}= 0.9$ in order to match unemployment dynamics in Germany after the introduction of the lockdown and short-time work scheme in March 2020.

Economic support measures are associated with a considerable increase in the governmental spending and, due to the mechanics of the tax rule, normally would trigger an upward adjustment of the tax rate. In order to avoid tax increases during the downturn, the adjustment of the tax rate is suspended during a lockdown.

#### Policy settings for the reproduction of German time series

The simulation output shown together with German data in Fig. 3 has been generated under a policy setting, in which 2 weeks after the appearance of the virus (corresponding to March 23, 2020) individual prevention measures, social distancing, working from home are introduced together with lockdown measures of intensity $\alpha ^l = 1$. These measures stay in place until the incidence value drops below $\beta ^o = 5$, at which point economic activities are fully resumed (after the adjustment period), i.e., $\alpha ^o = 0$. The parameter setting underlying these simulations is summarized in Table 4.

**Table 4:** Default values for lockdown policy

	Lockdown
Individual prevention measures	$\xi ^l = 0.625$
Social distancing	$n_{yy}^{p,l} = 2, n_{oo}^{p,l} = 0.5, n_{oy}^{p,l} = 1, n_{yo}^{p,l}= 0.5$
Working from home	Yes
Work contacts	$n^{w,l} = (4,5,8,2)$
Shopping contacts	$n^{s,l} = (5,20,10)$
Reduction in shopping frequency	$\Delta p^{s,l} = (0.1,0.33,0)$
Short time work	$\varphi = 0.7, \ q^{st} = 0.9$
Bailout	Yes
Lockdown intensity	$\alpha ^l = 1$
Incidence threshold where lockdown is lifted	$\beta ^o = 5$
Consumption reduction during opening	$\alpha ^o = 0$

## B: Additional results

In this appendix, we show that increasing the parameter $\beta ^o$ does not improve policy results compared to our default setting. In the main analysis in Sect. 5.2 we investigate the variation of $\beta ^l$, the threshold for activating the lockdown measures, but keep the threshold for leaving the lockdown at a relatively low constant value of $\beta ^o =5$. In particular, for policies with relatively high thresholds for entering the lockdown, also higher values of $\beta ^o$ could be considered. We explore the implications of such higher thresholds for our benchmark policy A (1, 0, 50) as well as for two other policies characterized by a high incidence threshold $\beta ^l = 50$, namely a policy with weak opening (E: (1, 0.5, 50) and a policy with weak lockdown (F: (0.5, 0, 50)). In Table 5, we show the effect of an increase in $\beta ^o$ under all three policies. The table shows that policies with higher values of $\beta ^o$ do not lead to better outcomes compared to our default value of $\beta ^o = 5$.

With respect to policy A, an increase in $\beta ^o$ gives rise to a trade-off between increasing mortality and decreasing GDP loss. However, comparing the values given in Table 5 with the effects of policies *C* and *E*, as depicted in Fig. 6, shows that all combinations of policy *A* with larger values of the threshold $\beta ^o$ are clearly dominated by these two policies both with respect to mortality and GDP loss.

**Table 5:** Variation in $\beta ^o$ for policies A, E and F

**A: (1,0,50)**				
$\beta ^o$	5	10	30	50
GDP loss [%]	3.73 (0.76)	3.98 (0.64)	3.56 (1.04)	3.24 (0.95)
Mortality [%]	0.077 (0.016)	0.086 (0.013)	0.112 (0.022)	0.134 (0.023)

**E: (1,0.5,50)**				
$\beta ^o$	5	10	30	50
GDP loss [%]	3.78 (0.38)	4.12 (0.4)	3.99 (0.44)	3.93 (0.41)
Mortality [%]	0.076 (0.022)	0.082 (0.019)	0.101 (0.022)	0.118 (0.027)

**F: (0.5,0,50)**				
$\beta ^o$	5	10	30	50
GDP loss [%]	1.29 (0.23)	1.25 (0.16)	1.53 (0.42)	1.25 (0.31)
Mortality [%]	0.144 (0.040)	0.127 (0.034)	0.121 (0.032)	0.141 (0.026)

## C: Policy phase-out under vaccination rollout

This appendix provides data on GDP loos, mortality, duration in lockdown, number of lockdowns, and public account deficit for the for policy scenarios A–D under vaccination rollout (Sect. 5.4). Containment measures have been fully terminated at the point in time, where 0%, 25%, 40%, and 100% of the population have had the opportunity to get vaccinated (Tables 6, 7, 8, 9).

**Table 6:** Comparison of policy results under vaccination rollout (0% case)

	A	B	C	D
$(\alpha ^l, \alpha ^o,\beta ^l)$	(1, 0, 50)	$(1,0,{\textbf {5}})$	$({\textbf {0.5}},0,{\textbf {5}})$	$({\textbf {0.5}},{\textbf {0.5}},{\textbf {5}})$
	*Benchmark policy*	*Low threshold*	*Weak lockdown*	*Cont. weak lockdown*
GDP loss [%]	1.78 (0.29)	2.04 (0.35)	0.45 (0.09)	0.81 (0.09)
Mortality [%]	0.215 (0.050)	0.138 (0.076)	0.209 (0.073)	0.210 (0.066)
Duration in lockdown	537.5 (120.1)	512.0 (188.1)	580.9 (159.1)	583.5 (128.7)
Number of lockdowns	0.94 (0.24)	3.66 (1.49)	2.90 (1.36)	1.84 (0.98)
Pub. Acc. Deficit [% of GDP]	2.27 (0.28)	2.33 (0.44)	1.65 (0.26)	1.42 (0.18)

Cells show means over 50 batch runs with standard deviation in brackets

**Table 7:** Comparison of policy results under vaccination rollout (25% case)

	A	B	C	D
$(\alpha ^l, \alpha ^o,\beta ^l)$	(1, 0, 50)	$(1,0,{\textbf {5}})$	$({\textbf {0.5}},0,{\textbf {5}})$	$({\textbf {0.5}},{\textbf {0.5}},{\textbf {5}})$
	*Benchmark policy*	*Low threshold*	*Weak lockdown*	*Cont. weak lockdown*
GDP loss [%]	2.27 (0.45)	2.49 (0.52)	0.67 (0.12)	1.09 (0.11)
Mortality [%]	0.062 (0.019)	0.031 (0.019)	0.051 (0.016)	0.050 (0.022)
Duration in lockdown	528.9 (138.0)	516.0 (172.9)	584.1 (144.1)	557.6 (162.3)
Number of lockdowns	0.92 (0.27)	3.74 (1.68)	3.04 (1.55)	1.68 (1.20)
Pub. Acc. Deficit [% of GDP]	3.12 (0.55)	2.91 (0.73)	1.93 (0.34)	1.57 (0.26)

Cells show means over 50 batch runs with standard deviation in brackets

**Table 8:** Comparison of policy results under vaccination rollout (40% case)

	A	B	C	D
$(\alpha ^l, \alpha ^o,\beta ^l)$	(1, 0, 50)	$(1,0,{\textbf {5}})$	$({\textbf {0.5}},0,{\textbf {5}})$	$({\textbf {0.5}},{\textbf {0.5}},{\textbf {5}})$
	*Benchmark policy*	*Low threshold*	*Weak lockdown*	*Cont. weak lockdown*
GDP loss [%]	2.61 (0.52)	2.77 (0.65)	0.80 (0.17)	1.25 (0.12)
Mortality [%]	0.066 (0.022)	0.024 (0.008)	0.038 (0.010)	0.035 (0.011)
Duration in lockdown	458.2 (180.9)	404.2 (205.0)	573.7 (150.5)	547.4 (183.3)
Number of lockdowns	0.92 (0.27)	4.30 (2.56)	2.40 (1.32)	1.62 (1.19)
Pub. Acc. Deficit [% of GDP]	3.68 (0.70)	3.18 (0.83)	2.20 (0.37)	1.72 (0.30)

Cells show means over 50 batch runs with standard deviation in brackets

**Table 9:** Comparison of policy results under vaccination rollout (100% case)

	A	B	C	D
$(\alpha ^l, \alpha ^o,\beta ^l)$	(1, 0, 50)	$(1,0,{\textbf {5}})$	$({\textbf {0.5}},0,{\textbf {5}})$	$({\textbf {0.5}},{\textbf {0.5}},{\textbf {5}})$
	*Benchmark policy*	*Low threshold*	*Weak lockdown*	*Cont. weak lockdown*
GDP loss [%]	2.93 (0.40)	3.07 (0.73)	1.20 (0.17)	2.03 (0.12)
Mortality [%]	0.064 (0.016)	0.023 (0.007)	0.035 (0.010)	0.035 (0.012)
Duration in lockdown	236.9 (41.2)	246.3 (71.9)	327.9 (50.8)	289.1 (86.6)
Number of lockdowns	0.98 (0.25)	5.14 (2.54)	3.28 (1.70)	1.48 (0.91)
Pub. Acc. Deficit [% of GDP]	3.92 (0.63)	3.41 (0.92)	2.43 (0.35)	2.12 (0.34)

Cells show means over 50 batch runs with standard deviation in brackets

## D: Statistical tests

This appendix provides the results from statistical tests. To verify the statistical significance for differences between points A, B, C and D in Fig. 6 and Table 1 in mortality and average GDP loss, we use the Mann–Whitney U test, a nonparametric test for unpaired samples. We document the *p* values in Tables 10 and 11, which are based on 50 batch runs. Table 12 shows the comparison between the three scenarios discussed in Sect. 5.3 documenting differences and *p* values. Tables 13, 14, 15 and 16 document the p-values of the comparison between policies A,B,C and D with respect to average GDP loss and mortility in the no-mutation and the higher infection scenarios.

**Table 10:** Mann–Whitney U test for GDP

Mutation scenario			
	B	C	D
$(\alpha ^l, \alpha ^o,\beta ^l)$	$(1,0,{\textbf {5}})$	$({\textbf {0.5}},0,{\textbf {5}})$	$({\textbf {0.5}},{\textbf {0.5}},{\textbf {5}})$
	*Low threshold*	*Weak lockdown*	*Weak cont. lockdown*
A			
(1, 0, 50)	$<0.0001$	$<0.0001$	$<0.0001$
*Benchmark policy*			
B		$<0.0001$	$<0.0001$
C			$<0.0001$

Cells show *p* values over 50 batch runs

**Table 11:** Mann–Whitney U test for mortality

Mutation scenario			
	B	C	D
$(\alpha ^l, \alpha ^o,\beta ^l)$	$(1,0,{\textbf {5}})$	$({\textbf {0.5}},0,{\textbf {5}})$	$({\textbf {0.5}},{\textbf {0.5}},{\textbf {5}})$
	*Low threshold*	*Weak lockdown*	*Weak cont. lockdown*
A			
(1, 0, 50)	$<0.0001$	$<0.0001$	0.0024
*Benchmark policy*			
B		$<0.0001$	$<0.0001$
C			0.1112

Cells show *p* values over 50 batch run

**Table 12:** Differences and Mann–Whitney U test between scenarios

Mutation versus no mutation scenario				
	A	B	C	D
$(\alpha ^l, \alpha ^o,\beta ^l)$	(1, 0, 50)	$(1,0,{\textbf {5}})$	$({\textbf {0.5}},0,{\textbf {5}})$	$({\textbf {0.5}},{\textbf {0.5}},{\textbf {5}})$
	*Benchmark policy*	*Low threshold*	*Weak lockdown*	*Cont. weak lockdown*
GDP [%]	2.42	2.21	0.83	$-0.19$
	$(<0.0001)$	$(<0.0001)$	$(<0.0001)$	$(<0.0001)$
Mortality [%]	0.028	0.012	0.035	0.042
	$(<0.0001)$	$(<0.0001)$	$(<0.0001)$	$(<0.0001)$

Mutation versus higher infection scenario				
	A	B	C	D
$(\alpha ^l, \alpha ^o,\beta ^l)$	(1, 0, 50)	$(1,0,{\textbf {5}})$	$({\textbf {0.5}},0,{\textbf {5}})$	$({\textbf {0.5}},{\textbf {0.5}},{\textbf {5}})$
	*Benchmark policy*	*Low threshold*	*Weak lockdown*	*Cont. weak lockdown*
GDP [%]	0.27	0.33	0.30	0.17
	$(<0.0001)$	$(<0.0001)$	$(<0.0001)$	$(<0.0001)$
Mortality [%]	$-0.043$	$-0.058$	$-0.075$	$-0.073$
	$(<0.0001)$	$(<0.0001)$	$(<0.0001)$	$(<0.0001)$

Cells show differences (mutation minus other scenario) and in brackets below *p* values for a comparison between the two pairs of scenarios. The upper part shows comparison for mutation versus no mutation, and the lower part versus higher infection scenario

**Table 13:** Mann–Whitney U test for GDP

No-mutation scenario			
	B	C	D
$(\alpha ^l, \alpha ^o,\beta ^l)$	$(1,0,{\textbf {5}})$	$({\textbf {0.5}},0,{\textbf {5}})$	$({\textbf {0.5}},{\textbf {0.5}},{\textbf {5}})$
	*Low threshold*	*Weak lockdown*	*Weak cont. lockdown*
A			
(1, 0, 50)	$<0.0001$	$<0.0001$	$<0.0001$
*Benchmark policy*			
B		$<0.0001$	0.0071
C			$<0.0001$

Cells show *p* values over 50 batch runs

**Table 14:** Mann–Whitney U test for mortality

No-mutation scenario			
	B	C	D
$(\alpha ^l, \alpha ^o,\beta ^l)$	$(1,0,{\textbf {5}})$	$({\textbf {0.5}},0,{\textbf {5}})$	$({\textbf {0.5}},{\textbf {0.5}},{\textbf {5}})$
	*Low threshold*	*Weak lockdown*	*Weak cont. lockdown*
A			
(1, 0, 50)	$<0.0001$	$<0.0001$	$<0.0001$
*Benchmark policy*			
B		0.0027	0.1099
C			0.1032

Cells show *p* values over 50 batch run

**Table 15:** Mann–Whitney U test for GDP

Higher infection scenario			
	B	C	D
$(\alpha ^l, \alpha ^o,\beta ^l)$	$(1,0,{\textbf {5}})$	$({\textbf {0.5}},0,{\textbf {5}})$	$({\textbf {0.5}},{\textbf {0.5}},{\textbf {5}})$
	*Low threshold*	*Weak lockdown*	*Weak cont. lockdown*
A			
(1, 0, 50)	$<0.0001$	$<0.0001$	$<0.0001$
*benchmark policy*			
B		$<0.0001$	$<0.0001$
C			$<0.0001$

Cells show *p* values over 50 batch runs

**Table 16:** Mann–Whitney U test for mortality

Higher infection scenario			
	B	C	D
$(\alpha ^l, \alpha ^o,\beta ^l)$	$(1,0,{\textbf {5}})$	$({\textbf {0.5}},0,{\textbf {5}})$	$({\textbf {0.5}},{\textbf {0.5}},{\textbf {5}})$
	*Low threshold*	*Weak lockdown*	*Weak cont. lockdown*
A			
(1, 0, 50)	$<0.0001$	0.0175	0.0250
*benchmark policy*			
B		$<0.0001$	$<0.0001$
C			0.5349

Cells show *p* values over 50 batch run

## Variables and Parameters

Table 17 provides a list of model variables and in Table 18 all parameters are listed together with their default values (which might differ between the four sectors).

**Table 17:** List of variables

Symbol	Description
Firms	
$A_i$	Labor productivity
$D_{i,w}$	Sum of daily sales in the previous sales cycle
$\hat{D}_{i,w}$	Demand expectation for the production and sales cycle starting in week *w*
$\textbf{F}_w$	Set of all private firms
$\textbf{F}_{k,w}$	Set of firms in sector *k*
$L_{i,w}$	Labor input in the production and sales cycle starting in week *w*
$\tilde{L}_{i,w}$	Planned labor input for the production and sales cycle starting in week *w*
$$ L^V_{i,w}$$	Open vacancies in week *w*
$$ L^R_{i,w}$$	Redundancies in week *w*
$$ P_{i,w}$$	Price in week *w*
$\Pi _{i,w}$	Profits of firm *i* in the previous production cycle
$Q_{i,w}$	Realized output in the production and sales cycle of week *w*
$\tilde{Q}_{i,w}$	Planned output for the production and sales cycle of week *w*
$$ S_{i,w}$$	Available liquidity in week *w*
$\tilde{S}_{i,w}$	Threshold liquidity level for dividends in *w*
$X_{i,t}$	Sales in period *t*
$Y_{i,t}$	Inventory stock available for sale in period *t*
$$ c_{i}$$	Unit costs
$$ c^F_{i}$$	Fixed costs
$d_{i,w}$	Dividends paid out by firm *i* to its shareholders in week *w*
$\mu _{i,w}$	Mark-up in week *w*
$n_{t}$	Number of firms at time *t*
$n_{k,w}$	Number of firms in sector *k* in week *w*
$$ s_{i,w}$$	Market share of firm *i* in week *w*
$$ w_{i}$$	Wage equal to sectoral wage $$w_k$$
Households	
$C_{h,w}$	Consumption budget
$\tilde{C}^S_{h,k,t}$	Intended consumption budget for sector *k*
$\mathcal {C}_{h,i,t}$	Desired quantity of product *i*
$$ C^S_{h,k,t}$$	Actual consumption budget for sector *k*
$$ E_{h,t}$$	Total expenditures in period *t*
$\textbf{H}_t$	Set of all households at time *t*
$\textbf{H}_t^Y$	Set of all young households
$\textbf{H}_t^O$	Set of all old households
$$ I_{h,w}^{Cap}$$	Capital income of a household
$I_{h,w}$	Total gross income of a household
$I^N_{h,w}$	Total net income of a household
$\bar{I}_{h,w}$	Smoothed average net income of a household
$W_{h,w}$	Wealth of a household
$$ m_t$$	Number of households at time *t*
$\omega _{h,w}$	Wage of household *h* in week *w*
$$ u_{h,w}$$	Unemployment benefits of household *h* in week*w*
$$ w^P$$	Level of pension
Labor market	
$\textbf{L}^S_{k,w}$	Set of workers forming the labor supply in sector *k*
$$ L^S_{k,w,}$$	Number of job seekers in sector *k*
$\textbf{L}^{HO}_{k,w}$	Set of workers in sector *k* that are eligible to work from home
$\textbf{U}_w$	Set of all unemployed households
$\textbf{U}^S_{k,w}$	Set of all unemployed households qualified for sector *k*
$\textbf{V}_{k,w}$	Set of all firms of sector *k* with open vacancies
$\textbf{W}^F_{i,w}$	Set employees of firm *i* in week *w*
$\textbf{W}^G_g$	Set of civil servants working for the public office *g*
$\textbf{W}^{HO}_{i,t}$	Set of workers able to work from home of firm *i* at time *t*
$\textbf{W}^{ST}_{i,t}$	Set of short time workers of firm *i* at time *t*
Goods market	
$\textbf{C}_{i,t}$	Set of clients of firm *i* at period *t*
$\textbf{C}_{k,t}$	Set of clients of a sectoral *k* mall at period *t*
${\Omega }_{h,k,t}$	Set of products of sector *k* considered for consumption choice of household *h*
Social Interactions	
$\textbf{CS}_{h,k,t}$	Set of co-shoppers of agent *h* in sector *k* at time *t*
$\textbf{CW}_{h,t}$	Set of co-workers of agent *h* at time *t*
$N^{a,a}_{h,t}$	Number of people met during social activities by agent *h* at time *t* divided per age group
$N^{c}_{h,k,t}$	Number of co-shoppers met by agent *h* while shopping in sector *k* at time *t*
$\bar{N}^{cs}_{h,k,t}$	Maximum number of co-shopper eventually met by agent *h* in sector *k* at time *t*
$N^{w}_{h,t}$	Number of co-workers met by agent *h* at time *t*
$\textbf{SA}^{a}_{h,t}$	Set of households belonging to a specific age-group met by agent *h* at time *t*
$\textbf{X}_{h,t}$	Set of colleagues of household *h* at time *t*
Government	
$\textbf{G}$	Set of all public sector offices
$$ GDP_w$$	Gross domestic product of the previous week
$$ L^P$$	Number of civil servants working for the government
$$ S^G_w$$	Public account
$$ T^C_w$$	Corporate tax revenues
$$ T^I_w$$	Income tax revenue
$\textbf{W}^S_{P,w}$	The set of civil servants working for the government
$\tau _w$	Tax rate
$\hat{\tau }_w$	Reference tax rate
$$ w^S_P$$	Wage paid in the public sector
Pandemic	
$\textbf{D}_t$	Set of deceased at time *t*
$\textbf{I}_t$	Set of actual infected at time *t*
$\textbf{I}^{inf}_t$	Set of infectious agents
$I^2_{h,t}$	Cumulative number of secondary infection caused by agent *h* at time *t*
$\textbf{R}_t$	Set of recovered at time *t*
$R_{0,t}$	Daily basic reproduction number
$R^{RKI}_{0,t}$	Robert Koch Institute reproduction number estimation
$\textbf{S}_t$	Set of susceptible at time *t*
$\textbf{T}_t$	Set of new infected between time *t* and $t+1$
$q^{a}_{t}$	Individual case fatality rate at time *t*
$\textbf{I}_t^{mut}$	Set of actual infected at time *t* with the mutation

**Table 18:** List of parameters

Symbol	Description	Value
Firms		
$[\bar{A}_k]$	Sector-specific average productivity	[97, 62, 48, 62]
$[\varphi _k]$	Target of firm liquidity relative to av. revenues during last 4 weeks	[1, 0.5, 0.5, 0]
$[\chi _{k}]$	Size of the sector-specific weekly inventory buffer	[0.0036, 0.0011, 0.0018, 0]
$[\delta _{k}]$	Sector-specific weekly depreciation rate of the inventory	[0.01, 1.00, 0.50, 0.00]
$[e_{k}]$	Estimated employment shares	[0.1170, 0.4362, 0.3268, 0.1200]
$\iota$	Production boost in case of stock-out	4
$[\lambda _{k}]$	Weekly fixed to variable cost ratio	[0.0752, 0.048, 0.048, 0.048]
$n_0$	Initial number of private firms	3780
$[\bar{\mu }_{k}]$	Upper bound firm mark-up	[0.18, 0.18, 0.18, 0]
$[\underline{\mu }_{k}]$	Lower bound firm mark-up	[0.25, 0.25, 0.25, 0]
$\rho ^D$	Firm demand expectation smoothing	0.5
$\zeta$	Dividend payout ratio	0.7
$q^{st}$	Probability that worker enters short-time work	0.9
Households		
$a^y_0$	Fraction of the young households	0.75
$[c_k]$	Fixed consumption quotas	[0.21, 0.50, 0.29]
$[p^{s}_k]$	Probability of shopping $k \in \{M,S,F\}$	[1, 1, 1]
$\eta$	Number of products for which a household collects prices at the mall	4
$\gamma ^{c}$	Intensity of consumer choice	16
$[h^{HO}_{k}]$	Sector proportion of home office workers	[0.45, 0.30, 0.00, 0.75]
$\kappa$	Adjustment wealth/income ratio	0.1/4
$m_0$	Initial number of households	100000
$[n^{w}_{k}]$	Work contact cardinality upper bound sector specific	[8, 8, 8, 8]
$[n^{c}_{k}]$	Shopping contact cardinality upper bound sector specific (manufacturing, service, food)	[10, 28, 10]
$[n^{p}_{a,a}]$	Cross-age contact cardinality upper bound yy,yo,oy,oo	[5, 2, 4, 2]
$\nu$	Wage replacement rate	0.60
$\Phi$	Target wealth/income ratio	32
$\phi$	Adjustment parameter consumption budget for essential product	0.01
$\rho ^{I}$	Income expectation smoothing	0.4
Government		
$n_P$	Number of public offices	600
*pen*	Pension as fraction of average wage	0.50
$\varphi$	Replacement rate of the short-time work program	0.7
$\rho ^T$	Adjustment speed of the tax rate	0.05
$\theta$	Fraction of public debt erased/added in one week	1/520
Pandemic		
$\delta _r$	Detection rate	0.15
$n^{icu}$	Number of intensive care units available per agent	$30*10^{-5}$
$p_{inf}$	Infection probability in a single contact	0.0725
$[\bar{q}^a_l]_{a = y,o}$	Individual fatality rate with under-utilization of ICU	[0.00099, 0.024]
$[\bar{q}^a_h]_{a = y,o}$	Individual fatality rate with over-utilization of ICU	[0.0027, 0.075]
$t_{0}$	Starting date of the pandemic	14
$t_{lnt}$	Latency period of the disease	5
$t_{inf}$	Infectious period of the disease	5
$t_{gen}$	Generation time	4
$\bar{t}_{rec}$	Maximum number of days being infected or recovery time	21
$t_{vac}$	Number of days after the pandemic for vaccine availability	561
$s_{vac}$	Maximum number of vaccination doses administered daily (vaccination speed)	337
$eff_{vac}$	Vaccine effectiveness	0.95
$eff_{vac}^{dec}$	Vaccine effectiveness decrease per day after administration	0.00343
$will_{vac}^o$	Willingness to become vaccinated (old HHs)	0.75
$will_{vac}^y$	Willingness to become vaccinated (young HHs)	0.75
$u^{icu}$	Fraction of infected people needing intensive care	0.01275
$\xi$	Reduction in infection probability coefficient default value	0
$t^{mut}$	Start of the mutation (only when activated)	162
$n^{mut}$	Number of agents initially infected by the mutation	3
$p_{inf}^{mut}$	Infection probability in a single contact for an agent infected by the mutation	$1.5 \ p_{inf}$

## Footnotes

1 To our knowledge, the only exception in this respect is Federico and Ferrari (2021), where the optimal lockdown policy of a social planner trying to minimize expected discounted social costs is characterized in the framework of a SIR model with a stochastic transition rate.

2 Incidence is measured as the reported number of newly infected over 7 days per 100.000 households.

3 These strategies have been called *the Hammer and the Dance* (Pueyo 2020) and have been found to be optimal also in other settings (Hellwig et al. 2020; Farboodi et al. 2020).

4 The model has been implemented in Julia, the code is open source and can be obtained from https://github.com/ETACE/ace_covid19.

5 In particular, we include the public sector in our model to capture that employment in this sector is not affected by lockdown measures.

6 Since our analysis focuses on a short time period (24 months) characterized by a severe economic crisis, we abstain from incorporating a market entry mechanism or productivity improvements into our model. Furthermore, we assume that the current crisis has no effect on the capital stock and hence do not explicitly incorporate a capital goods sector into our model.

7 Since our model does not include a representation of the credit market, credit relationships between firms, or a stock market, our analysis does neither capture the effects of the pandemic on the provision of credit nor the economic implications of bankruptcies on the financial system. Although these are certainly highly relevant issues, our focus here is on the real side of the economy, such that these considerations are beyond the scope of the paper. More precisely, the only effects of firm bankruptcy captured in our model are those on the labor market (employees lose their job and hence their wage income) and on the consumption goods market (fewer firms offering consumption goods).

8 Part of the services provided by the public sector are also healthcare services relevant for the treatment of (severe) cases of COVID-19 infections, but for the purpose of our analysis we do not explicitly distinguish between this part of the public sector and other services. As explained below, we take a reduced form approach for describing the capacity of the healthcare system, by assuming a maximum number of available intensive care unit beds for COVID-19 infected and calibrating this number from empirical data.

9 It should be noted that for the purpose of our model we take the number of available beds in intensive care units as given and constant (calibrated based on German data). Potential adjustments of the number of such beds and associated costs in the public sector are not considered since corresponding investments and training of medical staff do not seem to be feasible in the considered time frame.

10 See Appendix A.4 for more details.

11 It is assumed that in the absence of virus containment measures no employees work from home or are on short time work.

12 For example, Subramanian et al. (2021) estimate in a study based on data from New York City that the proportion of symptomatic COVID-19 cases ranges between 13% and 18%.

13 We assume that at most 50% of beds available in intensive care units can be used to treat COVID-19 patients.

14 See Table 4 in Appendix A.4 for a summary of the parameter settings underlying these simulation runs.

15 GDP is calculated on a weekly basis and the unit of measurement on the vertical axis in Fig. 4c is such that a constant flow of one unit throughout a year corresponds to an annual GDP per capita of 1000€. For the purpose of calculating the per capita GDP over time, we divide the current GDP with the population size at the beginning of our simulation. This avoids that the death of old agents, who do not work, induces an increase in per capita GDP. The only scenario where continuously adjusting the population size in the per capita GDP calculation would make any distinguishable difference to the results presented in the paper is that without any containment measures. In all other scenarios, the mortality is so small that such an adjustment would not matter.

16 In Basurto et al. (2020), it is shown in a slightly different setting that delaying the initial lockdown induces higher mortality without reducing the economic loss.

17 In addition, we assume that a lockdown is lifted once the incidence of newly infected falls below $\beta ^o =5$ for all policy scenarios. In Appendix B, we show that policies using larger values of $\beta ^o$ are dominated by those considered here.

18 Panel (b) of Fig. 7 shows that per capita GDP at the end of the runs, after the full recovery, on average is higher compared to the pre-pandemic level. The reason for this observation is a ’cleansing effect’ of the pandemic in the sense that production is moved from less productive to more productive firms, which increases average productivity. Although the bailout policy prevents a large wave of bankruptcies while it is in place, the reduction in demand and increased level of competition due to the virus containment policies implies that less productive firms lose market shares and partly cease to produce. About 10% of firms declare bankruptcy after the end of the bailout policy in month 18. As a result, more productive firms gain market shares.

19 In Basurto et al. (2020), we carry out a more detailed analysis of the effect and implications of the short-time work scheme and bailout program. In particular, we show there that the implementation of these programs not only reduces the GDP loss but also the public deficit accumulated during the duration of containment measures. Put differently, the negative implications for the public deficit of the higher unemployment and larger GDP loss emerging in the absence of these measures outweigh their direct costs for the public account.
